# Assessing Language Skills in Children Aged 4 to 6 Years with Autism Spectrum Disorder: A Prospective Study

**DOI:** 10.3390/children12121596

**Published:** 2025-11-24

**Authors:** Jade Mériaux, Sandrine Foin, Abdessadek El Ahmadi, Christine Assaiante, Pascale Colé

**Affiliations:** 1 Edouard Toulouse Hospital Center, 118 Chemin de Mimet, 13015 Marseille, France; 2 Psychology and Neuroscience Research Center, Mixed Research Unit 7077, Aix-Marseille University-National Center for Scientific Research, Saint-Charles Center, Pôle 3C, Case C, 3 Place Victor Hugo, 13331 Marseille, Cedex 03, France; sandrine.foin@etu.univ-amu.fr (S.F.); abdessadek.el-ahmadi@univ-amu.fr (A.E.A.); christine.assaiante@univ-amu.fr (C.A.)

**Keywords:** autism spectrum disorder, preschool, children, lexicon, comprehension, phonology, articulation, standardized test

## Abstract

**Background/Objectives:** Language impairments are highly prevalent in children with Autism Spectrum Disorder (ASD). In preschoolers (3–6 years), language development predicts future social outcomes. Despite the availability of standardized tests for typically developing children, few studies have specifically examined language impairments in preschool-aged children with ASD using these tools. This study aimed to comprehensively assess receptive and expressive lexicon, receptive comprehension, phonology and articulation using standardized tools, and to evaluate their feasibility. A secondary goal was to compare the results obtained with standardized tests to those from developmental batteries and hetero-assessments (caregiver reports). **Methods:** Forty-seven children with ASD aged 4 to 6 years participated. Assessments included standardized language tests, developmental batteries and hetero-assessments. The dichotomous Rasch model evaluated feasibility and item performance of standardized tests. Concordance across methods was analyzed via Pearson correlations and stepwise linear regression. **Results:** Standardized assessments were feasible for most participants despite wide variability in language abilities. Partial but non-equivalent concordance was found among assessment methods, each providing complementary insights into language profiles. **Conclusions:** Combining multiple assessment methods is crucial to capture the complexity of language development in children with ASD. Standardized tests can be adapted and provide more precise profiles than developmental batteries or hetero-assessments alone. A multimodal approach is essential to accurately identify language strengths and therapeutic targets in preschool-aged children with ASD.

## 1. Introduction

The Diagnostic and Statistical Manual of Mental Disorders, fifth Edition (DSM-5) [1] identifies language impairment as a potential indicator of Autism Spectrum Disorder (ASD), noting that receptive language is often more affected than expressive language. For language assessment in ASD, it recommends a multi-informant approach that includes clinical observations, developmental history and three main categories of assessment: standardized tests (normed on typically developing individuals), developmental batteries (which assess broad development domains and estimate developmental age) and informant-based measures, such as self-reports (when applicable) and third-party reports from caregivers or teachers (hetero-assessments). These same categories are frequently used in research contexts to assess language skills in children with ASD. However, the DSM-5 recommendations bring to light two major challenges. Firstly, the issue of consistency across different types of assessments must be addressed. Secondly, clinical practice faces a significant limitation due to the lack of standardized tools specifically adapted for assessing early language in children with ASD [2,3,4]—especially in the 4–6-year age group targeted by this study.

Language development in ASD is highly heterogeneous, ranging from a complete absence of verbal output to the use of complex sentences [5]. While qualitative communication deficits—in both verbal and nonverbal modalities (e.g., gestures, posture, eye gaze)—are common, quantitative speech delays (e.g., no single words by age 2, no two-word phrases by age 3; [6]) are not universal. Nevertheless, language difficulties are typically observable in around 80% of children with ASD by 18–24 months [7,8] and are often among the earliest concerns reported by caregivers [9,10]. Early language development in the preschool years (ages 3–6) is a robust predictor of later social functioning [8]. Delays during this period are associated with more pronounced autistic symptoms [11], whereas acquiring functional language by age 5—which encompasses phonological, lexical, morphosyntactic and discourse-level abilities in both expressive and receptive modalities—is associated with more favorable long-term outcomes [12]. This period is also critical as it coincides with entry into formal schooling and the onset of extensive social interactions, when language, literacy and numeracy skills develop rapidly [13]. Despite the availability of standardized tests for typically developing children, assessing language skills in preschool-aged children with ASD remains challenging. This is partly due to delayed diagnoses, which often occur between ages 3 and 6 [14,15], despite recommendations for identification by age 2 [16], and to the fact that core ASD symptoms peak in expression between ages 4 and 5 [17].

This study pursued several interrelated objectives. First, we aimed to assess the feasibility and reliability of administering standardized language assessments to children with ASD aged 4 to 6 years. Second, we examined the level of agreement between standardized tests and more commonly used tools in clinical and research contexts—namely, developmental batteries and hetero-assessments. We also explored the degree of concordance between developmental batteries and hetero-assessments. Direct assessments (standardized tests and developmental batteries) offer clear advantages over hetero-assessments: they provide objective, real-time observation of language behavior in controlled settings, thereby reducing biases related to environmental influences or caregiver interpretation. In particular, standardized language tests yield more precise and consistent data than developmental batteries [18]. Nevertheless, their use in ASD remains limited—rarely administered before age 3, but more commonly used only after age 7.

Systematic reviews and meta-analyses (e.g., [19,20,21,22]) report that studies of high-risk infants (i.e., siblings of children with ASD) tend to focus on early developmental windows (5–24 months), with ASD diagnoses typically confirmed between 24 and 36 months. These studies mostly rely on developmental batteries and hetero-assessments [23,24,25,26], while standardized language tests [27] are rarely employed. Language delays in both expressive and receptive modalities have been reported as early as 8 [28], 12 [29], 18 [30] or 36 months [31,32,33], and persist through ASD diagnosis at 36 months. However, cross-study comparisons are difficult due to significant heterogeneity in assessment tools, which often results in inconsistent findings [18]. These discrepancies may arise from floor effects [33] or the limited variability of early expressive language measures [31].

A major limitation of this body of research is the reliance on broad developmental tools that do not isolate specific language components—such as vocabulary or syntax—needed to distinguish between receptive and expressive language profiles [18]. While standardized tests offer age-normed scoring and greater specificity, developmental batteries often lack transparency in item selection, limiting the granularity of analysis. To our knowledge, only Yirmiya and colleagues [27] combined a developmental battery (Reynell Developmental Language Scales—RDLS; [34]) with a standardized test (Clinical Evaluation of Language Fundamentals—CELF—Preschool; [35]). They found lower expressive and receptive language in high-risk toddlers by 24 months, persisting to ASD diagnosis at 36 months. However, their reporting of only composite scores—without analysis of individual subtests—yielded a global profile comparable to that of a developmental battery alone, thereby precluding more detailed interpretation.

In older children and young adults with ASD (up to 19 years), standardized language assessments are more common [20], but this skews samples toward children with greater verbal abilities, potentially excluding the 25–30% with limited language [36,37,38]. Moreover, matching participants with ASD and those with typical development on nonverbal cognition can complicate interpretation. Findings across studies are mixed, depending on the measures and age groups studied: while some report preserved receptive and expressive abilities [39], others describe deficits in vocabulary, pronoun use or syntax [40,41]. Importantly, syntactic impairments appear to affect only a subgroup of children with ASD, some of whom exhibit profiles similar to developmental language disorder [42,43].

The present study sought to evaluate the suitability and reliability of standardized language assessments in children with ASD aged 4 to 6, a population that remains underrepresented in language assessment research [20]. To our knowledge, only two studies—by Kover and colleagues [18] and Thurm and colleagues [44]—have used standardized language tools in preschoolers with ASD. Both studies found greater impairments in receptive than expressive language, a pattern that diverges from typical development.

Kover and colleagues [18] specifically examined lexical development in 49 verbal children with ASD (ages 4–11), using the Peabody Picture Vocabulary Test, fourth edition (PPVT-4; [45]) for receptive and the Expressive Vocabulary Test, second edition (EVT-2; [46]) for expressive vocabulary. They found that both receptive vocabulary and nonverbal cognition were delayed relative to chronological age, and that receptive vocabulary had a slower developmental trajectory than expressive vocabulary—although this difference was reduced after controlling for nonverbal cognitive ability, suggesting that nonverbal cognition plays a key role in receptive language development.

Thurm and colleagues [44] assessed receptive and expressive language in 59 children with ASD using hetero-assessments at age 3, including the Vineland Adaptative Behaviour Scales (VABS; [47]) and the Sequenced Inventory of Communication Development (SICD)-parent report [48]. At age 5, language assessment was conducted using standardized tests, including the Verbal comprehension and Naming vocabulary subtests of the Differential Abilities Scales (DAS; [49]), or alternatively, depending on the child’s developmental level, a developmental battery, either the Infant MSEL [50], or the MSEL [51]. They found that early communication skills and nonverbal Intellectual Quotient (IQ) at age 3 predicted both expressive and receptive language outcomes at age 5, with ASD diagnosis predicting poorer receptive language, aligning with observed lower scores.

Importantly, our study also examined the concordance between standardized language tests, developmental batteries and hetero-assessments. To date, only Thurm and colleagues [44] combined these assessment approaches in a sample of preschool-aged children with ASD. They investigated the predictive value of Communication and Socialization subscale scores from the VABS at age 3 on verbal and language outcomes measured by the DAS and the MSEL at age 5. Results showed that early communication skills significantly predicted both expressive and receptive language development at age 5, whereas socialization skills predicted only receptive language development. However, while informative, Thurm and colleagues’ study [44] did not examine the concordance of receptive and expressive language scores across different assessment methods. Nor did it explore the consistency of specific linguistic domains—such as receptive and expressive vocabulary, verbal comprehension, phonology and articulation—across standardized tests, developmental batteries and hetero-assessments.

Importantly, both studies converged in identifying language difficulties in ASD using standardized tests. However, these conclusions are drawn from only two studies—one of which included participants older than preschool age. Furthermore, comparison between the two studies is complicated by the use of different assessment tools.

Thus, our study addresses two primary research questions: (1) Can standardized tests normed on typically developing children to evaluate various language skills (receptive and expressive lexicon, receptive comprehension, phonology, articulation) reliably assess children ASD aged 4 to 6 with severe language impairments, a population with limited standardized language assessment research? This age group is particularly understudied, in part because 50–74% of preschoolers with ASD have minimal or no functional verbal language [52]. Nonetheless, despite the challenges in assessing language skills in this population, language difficulties are widely regarded as a core and reliable marker of ASD [44]. (2) To what extent are results consistent across standardized language tests, developmental batteries and hetero-assessments? In light of its limited sample size and exploratory design, this investigation should be regarded as a prospective, hypothesis-generating study, intended to inform and refine future research directions and clinical assessment practices.

## 2. Materials and Methods

### 2.1. Participants

The study involved 47 French preschool-aged children with ASD (36 boys, 11 girls, *M* = 5 years 9 months ± 8 months, age range = 4 years 0 months to 6 years 9 months). They were recruited locally through various medical and school institutions located in the Provence-Alpes-Côte d’Azur and Auvergne-Rhône-Alpes regions: Saint-Louis Early Medico-Social Action Center, Saint-Jérôme Day Hospital and Pythéas Medico-Psychological Center—Edouard Toulouse Hospital Center (Marseille); Salvator Polyvalent Early Medico-Social Action Center—Salvator Hospital (Marseille); North Early Medico-Social Action Center—North Hospital (Marseille); La Bricarde Autism Nursery School Unit, Early Medico-Social Action Center and Jean Maurel Autism Nursery School Unit—Regional Association for the Integration of people with disabilities or in difficulty (Marseille, Orange and Puyricard); Florian Departemental Medico-Psycho-Pedagogical Center—General Council of Bouches-du-Rhône (Marseille), Les Tamaris Autism Nursery School Unit—Association of Parents of Disabled Children (Bollène), Aiguebelle Autism Nursery School Unit—Association of Pupils of Public Education Sud-Rhône-Alpes (Donzère), Early Medico-Social Action Centers—Union for the Management of Health Insurance Fund Establishments (Hyères and Toulon), Louis Pécout Autism Nursery School Unit—Regional Association for the Education and Placement of Handicapped Young People (La Ciotat) and Early Medico-Social Action Center—George Sand Hospital Center (La Seyne-sur-Mer). Our study did not include a control group because we used standardized tests with established norms that provide comparative metrics. Moreover, our research questions focused on evaluating the appropriateness and reliability of standardized language assessments, as well as the concordance between language assessment tools within the ASD population, rather than on comparisons between groups.

Approval for the study was granted by the South Mediterranean I Personal Protection Committee under the reference 2020-A02980-39 on 2 December 2020. Before taking part in the study, at least one parent of each participant reviewed the written information sheet together with the examiner and signed a statement of non-opposition.

Children were assessed in their regular care environment and were included if they had received a diagnosis of ASD from the referring physician based on the criteria outlined in the DSM-5 [1].

In addition to the diagnosis, children were required to have undergone a comprehensive assessment that included a hetero-assessment using the VABS, second edition (VABS-II; [53]) and a direct developmental assessment—such as the Psychoeducational Profile, third edition (PEP-3; [54])—or a cognitive evaluation adapted to each child’s profile.

Children under the age of 4 were not included in the study, since an official ASD diagnosis is rarely made before this age and because the inclusion criteria required both a formal diagnosis and a comprehensive evaluation.

The VABS-II is a semi-structured interview conducted with a parent or caregiver, and it evaluates adaptive functioning in four areas such as communication, daily living skills, socialization and motor skills. The PEP-3 evaluates developmental level and delays in children with developmental disorders, aged from 2 to 7 and a half years. It covers both developmental and maladaptive behavioral areas, including verbal and nonverbal cognition, language, motor skills, oculomotor imitation, affective expression, social reciprocity, and characteristic motor and verbal behaviors.

Descriptive characteristics of the sample are reported in Supplementary File S1 (Table S1).

Four children in the sample had been diagnosed with asthma and were undergoing treatment. One child had been diagnosed with Attention Deficit Hyperactivity Disorder (ADHD). Specifically, 39 children were receiving speech therapy. 35 children had individualized human support at school and 11 children were enrolled in a specialized Autism Nursery School Unit.

### 2.2. Measures

#### 2.2.1. Hetero-Assessments with the Parent

Parents completed the VABS-II and the French inventories of communicative development (or Inventaires Français du Développement Communicatif, IFDC; [55]) The IFDC, adapted from the MacArthur Communicative Development Inventories [56], were completed retrospectively to assess early communicative development, specifically communicative gestures and vocabulary skills at 12, 18 and 24 months. Part 1 (for 12 months) evaluates babbling, the production of 25 gestures and the comprehension or production of 81 words. Part 2 (for 18 months) focuses on the comprehension or production of 97 words and on word combinations. Part 3 (for 24 months) assesses the production of 100 words and the average sentence length, but this last measure was excluded because no child exceeded 2.66 words. Raw scores were used for analysis instead of percentile ranks.

#### 2.2.2. Developmental Battery

Children were assessed with the PEP-3, which provided a direct measure of developmental functioning (see Section 2.1).

#### 2.2.3. Standardized Tests

All standardized tests used in this study align with French clinical recommendations [16,57,58]. Accordingly, we selected the French adaptation of the PPVT (or Echelle de Vocabulaire en Images Peabody, EVIP; [59]), the syntaxico-semantic comprehension test (or Epreuve de COmpréhension Syntaxico-SEmantique, E.CO.S.SE; [60]) and the oral language assessment 2–6 years old (or Evaluation du Langage Oral 2–6 ans, EVALO 2–6; [61]) to maximize construct coverage, psychometric robustness in French and comparability with prior ASD research. EVIP and E.CO.S.SE are explicitly recommended and offer robust French-language norms [16]. Notably, the EVIP and the E.CO.S.SE are, respectively, the French adaptations of the PPVT-Revised (PPVT-R; [62]) and the Test for the Reception of Grammar (TROG; [63]), and PPVT and TROG versions are widely used in international research [20,22]. Moreover, the PPVT-R and the EVIP were constructed using Rasch item-response modeling to calibrate item difficulty across the developmental continuum. This approach orders items with respect to age and supports the definition of age-appropriate administration bands (basal and ceiling rules), thereby improving the precision and interpretability of the norms. EVALO 2–6 is relatively recent and affords a comprehensive assessment of both receptive and expressive oral language skills [57].

##### Receptive Vocabulary

Receptive lexicon was assessed using the EVIP and the Designation from a cue subtest from the EVALO 2–6.

The EVIP assesses lexical breadth in children aged 2 years and 6 months to 18 years. The child points to one of four images that matches a spoken word, with the three remaining images serving as distractors: a phonological distractor (sharing sounds with the target word), a semantic distractor (related in meaning to the target word) and an unrelated distractor (having no relationship to the target word). Correct answers score 1; scoring stops after 6 errors in 8 consecutive items. The raw score corresponds to the total number of correct answers, with a possible maximum score of 170. In our sample, the highest item reached was 65, item 65 being the last to obtain at least one correct response. This raw score is then converted into a standardized score based on the child’s age, with a mean of 100 and a Standard Deviation (SD) of 15. 46 children completed this test.

We also administered the Designation from a cue subtest from the EVALO 2–6 language assessment battery. This battery includes two versions: a «young child» version (for ages 2 years 3 months to 4 years 3 months) and an «old child» version (for ages 4 years 3 months to 6 years 3 months). In our sample, 5 children were assessed using the «young child» version, 37 children using the «old child» version and 5 older children were not assessed with this tool. The Designation from a cue subtest requires children to identify and point to images based on a semantic or taxonomic category provided by the experimenter (e.g., «Show me the animal») and offers an assessment of lexical breadth as well as insight into children’s lexical organization. Correct answers score 1; no formal stopping rule but, to prevent the child from experiencing repeated failure, testing was stopped after 5 consecutive errors. The raw score (maximum 22) was converted to z-scores (*M* = 0, SD = 1).

##### Expressive Vocabulary

Expressive lexicon was assessed through the Denomination subtest shortlist from the EVALO 2–6 battery, which requires the child to name pictures. This subtest provides, for each word presented to the children, a procedure that simultaneously evaluates both word knowledge (Denomination-Lexicon scoring) and phonological representation quality (Denomination-Phonology scoring) through two distinct scoring systems. The lexical scoring system (Denomination-Lexicon) provides immediate and precise evaluation of word retrieval accuracy and speed. For instance, if the target word is «chemise» ([shirt]), the child might produce the correct phonological form or a phonologically incorrect variant, such as «temise» (nonword). If the child’s response is incorrect—whether it is a semantically related word like «veste» ([jacket]), an unrelated word like «table» or no response at all—the examiner provides a phonemic cue by giving the initial phoneme of the target word and prompts the child to try again. This procedure helps distinguish between difficulties in retrieving words from memory and a true lack of word knowledge. Responses are scored as 2 for a correct initial answer, 1 for a correct answer after oral assistance and 0 for an incorrect response. Raw scores reflect the total number of correct answers, with a maximum of 80 for initially correct responses and 40 for correct responses following assistance.

The phonological scoring system (Denomination-Phonology) assesses the phonological accuracy of the produced word. This procedure distinguishes between naming errors resulting from deficits in word knowledge and those stemming from phonological articulation difficulties. When the child makes an error during picture naming—either producing a word with incorrect phonology, failing to produce the expected target or producing no word, the examiner offers the first phoneme as a cue and asks the child to name the image; if phonological distortions occur, the examiner models the correct word and prompts the child to repeat it. An initially correct response or one given with oral assistance scores 2, a correct response after repetition scores 1 and an incorrect response scores 0. Raw scores correspond to the total correct answers, with maximum possible scores of 80 (initial or assisted response) and 40 (response after repetition). Although there is no formal stopping rule, the test was stopped after 5 consecutive errors to avoid repeated failure. Raw scores were converted into age-based z-scores (*M* = 0, SD = 1).

##### Oral Comprehension

Oral language comprehension was evaluated using the Understanding of topological terms subtest from the EVALO 2–6 and the E.CO.S.SE.

In the Understanding of topological terms subtest, the child is asked to place a dog figurine relative to a bench based on a spatial instruction from the examiner, such as «put the dog on the bench». Each correct response is scored 1. Although there is no formal stopping rule, the test was ended after 5 consecutive errors to avoid repeated failure. The maximum raw score varies by version: 6 for the «young child» and 9 for the «old child» version. Raw scores are then converted into age-based z-scores with a mean of 0 and a SD of 1.

The E.CO.S.SE evaluates oral sentence comprehension in children aged 4 to 12 years. The child is presented with an oral word or sentence (e.g., «le garçon court» [the boy runs] or «la balle est rouge» [the ball is red]) and must select the matching image from four options. In our sample, 46 children were assessed using this tool. Each error scores 1. Although the author suggests stopping the test after 6–7 failed blocks (24–28 items), we ended it after 5 consecutive errors to avoid repeated failure. The raw score is the number of errors (maximum 92) and is converted into a z-score adjusted for age, gender and parental socioeconomic status (*M* = 0, SD = 1). In our study, we used the number of correct responses as the raw score.

##### Articulation Skills

Articulation skills were assessed with the Orofacial and lingual praxis subtest from the EVALO 2–6. The child had to imitate bucco-facial movements (e.g., tongue out, lips pursed, cheeks puffed, blowing) and sound-based actions (e.g., phonemes, prolonged kissing sound, tongue click) demonstrated by the examiner. Each correct response scored 1, and incorrect responses scored 0. Although there is no formal stopping rule, the test was ended after 5 consecutive errors to avoid repeated failure. The raw score (maximum 18) was converted into a z-score adjusted for age (*M* = 0, SD = 1).

### 2.3. Procedure

An initial two-hour session was held with the parent to explain the study, review the written information sheet, obtain signed non-opposition consent and administer the IFDC. It was followed by an average of three weekly sessions with the child, each lasting approximately 1.5 h, during which the comprehensive language assessment protocol was administered. The number and duration of sessions were adapted to the child’s availability. All sessions were conducted either by the principal investigator, a psychologist specialized in neuropsychology, or by trained graduate students in neuropsychology or neuroscience under the investigator’s supervision. Graduate students completed structured preparation prior to data collection, including: (1) manual-based familiarization and practice administrations for each test, (2) direct observation of the principal investigator conducting assessments, and (3) ongoing supervision meetings throughout the study to ensure procedural fidelity and scoring reliability. Each parent–child dyad was typically assessed by the same examiner across sessions. To optimize child engagement and data validity, assessments followed a flexible, child-centered sequence tailored to each participant’s cooperation and fatigue. Because language measures are cognitively demanding, they were introduced only after achieving the best feasible level of child engagement. Although test order was individualized across sessions, all children completed the same set of assessments (age-appropriate forms), ensuring comparability of data despite individualized administration sequences.

### 2.4. Overview of Statistical Analysis

The feasibility of using standardized language tests—normed on typically developing populations—with children aged 4 to 6 years with ASD was first assessed with an item-level analysis using the dichotomous Rasch model [64,65]. This model, based on binary scoring (1 for correct, 0 for incorrect), estimates item difficulty to characterize the relationship between individual ability and the probability of a correct response. According to the Rasch model, the likelihood of a correct answer is determined by the difference between item difficulty and individual skill. Thus, individuals with higher language abilities are more likely to succeed across all items, while easier items are generally answered correctly by most participants. The Wright map provides a visual representation of participant ability and item difficulty along a common scale: participants are ranked by ability on the left and items are ordered by difficulty on the right. Higher positions on the continuum reflect stronger language skills (for participants) or greater difficulty (for items), whereas lower positions indicate lower ability or easier items. The symmetry of the item distribution offers insight into the overall difficulty of the test. This analytical approach helps evaluate the appropriateness of test items (i.e., whether they are neither too easy nor too difficult), and allows detection of potential ceiling or floor effects as well as the overall psychometric quality of the tests. Goodness-of-fit statistics—with acceptable mean square values between 0.7 and 1.3 for our sample [66]—were used to evaluate how well each item fits the underlying construct. Higher fit statistics suggest increased response variability [67]. Person-separation reliability (with values approaching 1 indicating better discrimination) reflects the test’s capacity to distinguish among participants with different levels of ability and supports the construct validity of the assessments [67,68,69].

Then, to examine how the different language components assessed in this study relate to one another and to gain a more integrated understanding of children’s overall language competence, a series of Pearson correlation analyses was conducted on Rasch scores across the seven language variables. Particular attention was given to the relationship between receptive and expressive lexical abilities, in light of previous findings indicating greater challenges in receptive language among children. To further investigate this discrepancy, paired Student’s *t*-tests were used to compare performance in receptive versus expressive lexical domains based on Rasch scores.

Finally, to assess the concordance between standardized tests, developmental battery and hetero-assessment measures, we first conducted Pearson correlation analyses. Subsequently, stepwise linear regression analyses were conducted to assess the level of agreement between the assessment methods by identifying the combination of predictors that best accounted for variance in each language component, thereby quantifying the degree of overlap among the tools.

## 3. Results

All data analyses were conducted using the statistical softwares Jamovi, version 2.3.28, JASP, version 0.19.1.0 and R, version 4.4.2.

### 3.1. Q1: Can Standardized Tests Normed on Typically Developing Children Reliably Assess the Language Skills of Children Aged 4 to 6 with ASD?

#### Rasch Analysis

**Figure 1:**
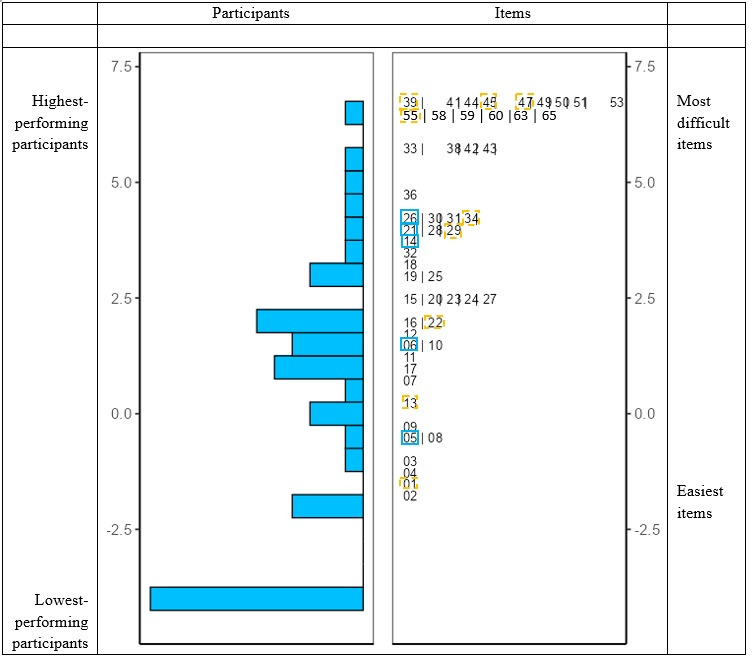
Wright map of the EVIP. Orange (dashed line): item infits < 0.7; blue (solid line): item infits > 1.3.

To enhance clarity and conciseness in the presentation of results, we reported Rasch analyses only for the EVIP in the main text. Complete Rasch analyses for the remaining language assessments are available in Supplementary File S2 (Figures S1–S8). The person reliability value was high (0.910, *p* < 0.001), indicating that the test reliably measured the intended construct. The majority of item infits fell within the acceptable range [0.7; 1.3], with 9 items < 0.7 and 5 items > 1.3, suggesting adequate model fit. As shown in Figure 1, there was limited overlap between participant ability and item difficulty distributions: 12 out of 46 participants scored 0 on the test (26%) and 12 out of 65 items were not answered correctly by any participant (and were thus excluded from the Rasch analysis). Despite these floor effects, the Wright map revealed a relatively good match between item difficulty and participant ability for the remaining 74% of the sample. The participant distribution on the left side showed that, while a substantial subgroup clustered at the lowest ability level, the other 74% displayed an approximately normal distribution with a slight positive skew across the ability continuum. The item distribution on the right side demonstrated a reasonable spread of difficulty levels—from easier items (Rasch values around −2.5) to more challenging ones (values around 7.5)—that aligned well with this range of participant abilities. These findings suggested that, while the EVIP may not adequately capture the abilities of the lowest-performing children with ASD, it provided meaningful and precise measurement for the majority of participants in this age range, supporting its efficiency for assessing receptive vocabulary skills in children with ASD aged 4 to 6 with emerging language skills.

Excluding the 12 participants who scored 0, the distribution function of receptive vocabulary was approximately normal, with a slight positive skew, suggesting that most children were able to respond variably to the items. The Rasch analysis indicated that the EVIP was appropriately calibrated for 74% of participants, who demonstrated differentiated responses. However, for the remaining 26%, the test proved to be too challenging (lowest negative score on the Rasch scale).

Moreover, the analysis confirmed that items clustered at similar levels on the Wright map were of comparable difficulty within our sample, suggesting that the test could be shortened by retaining a few representative items per level without significant loss of psychometric information. Finally, the item difficulty, as determined by chronological age norms, did not always correspond to observed performance in our atypical sample—highlighting that developmental trajectories in children with ASD may diverge from typical patterns.

A summary of key Rasch outcomes across all tests is provided in Table 1.

**Table 1:** Summarize of key findings from Rasch analyses.

			Person Reliability	Item Infits < 0.7	Item Infits > 1.3	Items Failed by All Participants	Participants Scored 0	Score Distribution
Lexicon	Receptive lexicon	EVIP	0.910 ***	9	5	12	12 (26%)	Approximately normal with a slight positive skew
		Designation from a cue	0.796 ***	2	1	0	15 (37%)	Uniform
	Expressive lexicon	Denomination-Lex 1	0.700 ***	4	3	3	22 (52%)	Globally uniform
Receptive comprehension		Understanding of topological terms	0.619 ***	5	0	0	14 (34%)	Positive skew
		E.CO.S.SE	0.915 ***	27	6	19	12 (26%)	Approximately normal with a slight positive skew
Phonology		Denomination-Phono 1	0.733 ***	4	1	1	21 (50%)	Globally uniform
Articulation		Orofacial and lingual praxis	0.816 ***	2	0	0	10 (24%)	Approximately normal with a slight negative skew

*** *p* ≤ 0.001.

### 3.2. Q2: To What Extent Are the Results of Standardized Language Tests Consistent with One Another, as Well as with Scores from Developmental Batteries and Hetero-Assessments?

#### 3.2.1. Pearson Correlation Analyses

##### Inter-Correlation Matrix of Rasch Scores on Language Tests

Pearson correlation analyses were conducted using Rasch scores rather than standardized scores because they provide objective estimates that are independent of item difficulty (for individuals) and individual ability (for items). This makes them a more robust metric for examining relationships among constructs. The full inter-correlation matrix is presented in Supplementary File S3 (Table S2).

Rasch scores from language assessments were significantly and positively correlated with one another, with coefficients ranging from *r* = 0.450 ** to *r* = 0.911 ***. One exception was the Orofacial and Lingual Praxis subtest, which showed only moderate correlations with the Denomination-Lex 1 and Understanding of topological terms subtests (respectively, *r* = 0.450 ** and *r* = 0.477 **). These more moderate correlations suggest that orofacial praxis may reflect partially distinct abilities from those assessed by language-based measures. Notably, the very strong correlation between Denomination-Lex 1 and Denomination-Phono 1 (*r* = 0.911 ***) subtests (which represented alternative scoring methods applied to the same subtest) indicated that they likely captured overlapping underlying constructs. This redundancy suggests that retaining both may be unnecessary and that either score could be selected depending on the specific focus of the assessment.

##### Focus on Receptive and Expressive Lexicon

Paired-sample *t*-tests were conducted to compare receptive and expressive lexicon performance. As shown in Table 2, expressive lexicon performance—measured by Denomination-Lex 1—was significantly lower than receptive lexicon performance, as assessed by both the EVIP (*t*(40) = −3.11, *p* < 0.01) and the Designation from a cue subtest (*t*(40) = −2.21, *p* < 0.05). Children demonstrated poorer performance on the expressive lexicon test. Thus, the expressive lexicon test can be considered more challenging than the receptive one.

**Table 2:** Paired-sample *t*-tests comparing receptive and expressive lexicon assessments using Rasch scores.

Lexicon	Test	Mean	SD	*t*(df = 40)	*p* Value	Mean Differences	
						Mean	SD
Receptive lexicon	EVIP	−0.006	2.93	−3.11 **	0.003	−1.018	0.327
	Designation from a cue	0.455	2.27	−2.21 *	0.033	−0.557	0.252
Expressive lexicon	Denomination-Lex 1	1.012	2.12				

df (degrees of freedom). ** *p* ≤ 0.01; * *p* ≤ 0.05. A negative *t*-value indicates lower scores on the expressive test relative to the receptive one.

##### Inter-Correlation Matrix Between Hetero-Assessment and Developmental Battery Scores

For the purpose of Pearson correlation analyses between Rasch scores on language tests and scores from hetero-assessments and developmental batteries, only the subdomains specifically targeting language skills were retained. These included the v-scale scores (standardized scores with a mean of 15 and a SD of 3) for the Receptive and Expressive language subdomains of the VABS-II. From the IFDC, we selected raw scores corresponding to the number of words understood and/or produced at 12 and 18 months, and the number of words produced at 24 months. From the PEP-3, we used the developmental age scores for the Receptive and Expressive language subdomains.

The complete inter-correlation matrix is presented in Supplementary File S3 (Table S3).

Results showed that most scores derived from the IFDC and the VABS-II, both hetero-assessment tools, were not significantly correlated. An exception was observed for the Receptive language subdomain of the VABS-II, which showed a significant moderate positive correlation with the number of words understood on the IFDC at 18 months (*r* = 0.397 **). In contrast, the Receptive and Expressive language subdomains of the PEP-3 were positively correlated with nearly all hetero-assessment measures (*r* values ranging from *r* = 0.357 * to *r* = 0.684 ***), with the exception of: (a) the number of words understood at 12 months on the IFDC, and (b) the correlation between the Receptive language subdomain of the PEP-3 and the number of words understood at 18 months on the IFDC.

##### Inter-Correlation Matrix Between Rasch Scores on Language Tests and Hetero-Assessment Scores

The inter-correlation matrix between Rasch scores on language tests and hetero-assessment scores is presented in Supplementary File S3 (Table S4).

No significant correlations were observed between any of the IFDC scales and the standardized language tests. In contrast, all language tests—except for the Understanding of topological terms subtest—were significantly and moderately positively correlated with the Receptive language subdomain of the VABS-II (*r* values ranging from *r* = 0.344 * to *r* = 0.464 **). Notably, the correlation between the Orofacial and Lingual Praxis subtest and the Receptive language subdomain of the VABS-II was strong (*r* = 0.542 ***). Furthermore, all language tests demonstrated significant and strong positive correlations with the Expressive language subdomain of the VABS-II (*r* values ranging from *r* = 0.541 *** to *r* = 0.753 ***).

##### Inter-Correlation Matrix Between Rasch Scores on Language Tests and Developmental Battery Scores

The inter-correlation matrix between Rasch scores on language tests and the PEP-3 developmental battery scores is presented in Supplementary File S3 (Table S5).

All language tests showed significant positive correlations with the Receptive language subdomain of the PEP-3 (*r* values ranging from *r* = 0.504 ** to *r* = 0.751 ***), with the exception of the Designation from a cue and Understanding of topological terms subtests, which showed moderate correlations (respectively, *r* = 0.414 * and *r* = 0.457 *). In terms of expressive language, the EVIP, the Denomination-Lex 1, as well as the E.CO.S.SE were strongly and significantly correlated with the Expressive language subdomain of the PEP-3 (*r* values ranging from *r* = 0.561 *** to *r* = 0.773 ***). However, the Designation from a cue and Orofacial and lingual praxis subtests showed only moderate correlations with the Expressive language subdomain of the PEP-3 (respectively, *r* = 0.386 * and *r* = 0.466 *). No significant correlation was observed between the Understanding of topological terms subtest and the Expressive language subdomain of the PEP-3.

#### 3.2.2. Stepwise Linear Regression Analyses

Stepwise linear regression analyses were conducted to identify concurrent predictors of children’s language skills across lexicon, receptive comprehension, phonology and articulation components. This approach was used to assess the concordance between standardized language tests, developmental battery and hetero-assessments. The degrees of freedom varied across analyses due to differences in sample size stemming from age restrictions or missing data.

From two complementary perspectives, this analysis addresses the following research questions: (a) To what extent do scores from hetero-assessments and developmental battery predict performance on standardized language tests? In other words, can hetero-assessments alone suffice or is direct assessment still necessary? (b) Conversely, do Rasch scores from standardized language tests predict results obtained from hetero-assessments and developmental battery? That is, are standardized tests sufficiently reliable to predict subjective and informant-based measures? To explore these questions, we performed two sets of stepwise regressions. First, we treated standardized test Rasch scores as dependent variables with hetero-assessment and developmental battery scores as predictors (addressing question a). Next, the roles were reversed to address question (b).

The results presented below focus on question (a). Here, Rasch scores from standardized language assessments were included as dependent variables, while hetero-assessment and developmental battery scores served as predictors. This analysis tests whether data from standardized tests can be effectively approximated or replaced by information from hetero-assessments and developmental battery, thereby evaluating the necessity of direct assessment.

##### Stepwise Linear Regression Predicting Rasch Scores on Language Tests from Hetero-Assessment and Developmental Battery Scores

The resulting regression models are summarized in Table 3. Detailed statistical outcomes are available in Supplementary File S4.

As shown in Table 3, regression analyses revealed that a single-step model significantly predicted performance on lexical breadth (EVIP), with PEP-3 Receptive language accounting for 43% of the variance. A one-step model also significantly predicted performance on word retrieval accuracy and speed (Denomination-Lex 1 scoring), with PEP-3 Expressive language accounting for 52% of the variance. Lexical breadth and organization (Designation from a cue subtest) were significantly predicted by a one-step model with PEP-3 Expressive language accounting for 19% of the variance. Oral comprehension of topological terms (Understanding of topological terms subtest) was significantly predicted by a one-step model with PEP-3 Receptive language accounting for 20% of the variance. Oral sentence comprehension (E.CO.S.SE) was significantly predicted by a two-step model including PEP-3 Expressive language and VABS-II Receptive language, which together explained 65% of the variance. Phonological accuracy of the produced word (Denomination-Phono 1 scoring) was significantly predicted by a two-step model including PEP-3 Receptive language and VABS-II Expressive language, jointly accounting for 62% of the variance. Finally, articulation skills (Orofacial and lingual praxis subtest) were significantly predicted by a one-step model with VABS-II Receptive language accounting for 31% of the variance.

The following analysis addresses question (b). In this model, scores from the hetero-assessments and developmental batteries were entered as dependent variables, while Rasch scores from the standardized language tests served as predictor variables.

##### Stepwise Linear Regression to Explain Receptive Language Scores from Hetero-Assessments and Developmental Battery Using Rasch Scores from Receptive Language Standardized Tests

The linear regression models are summarized in Table 4, with detailed results available in Supplementary File S4.

As shown in Table 4, a one-step regression model significantly predicted VABS-II Receptive language, with a single predictor—E.CO.S.SE (oral sentence comprehension)—accounting for 19% of the variance. Similarly, a one-step regression model significantly predicted PEP-3 Receptive language, with E.CO.S.SE again emerging as the sole predictor, explaining 53% of the variance.

##### Stepwise Linear Regression to Explain Expressive Language Scores from Hetero-Assessments and Developmental Battery Using Rasch Scores from Expressive Language Standardized Tests

The linear regression models are presented in Table 5, with detailed results available in Supplementary File S4.

As shown in Table 5, a two-step regression model significantly predicted VABS-II Expressive language, with two predictors—Denomination-Lex 1 (word retrieval accuracy and speed) and Orofacial and lingual praxis (articulation skills) subtests—accounting for 66% of the variance. Similarly, a two-step regression model significantly predicted PEP-3 Expressive language, with the same two predictors accounting for 60% of the variance in performance.

## 4. Discussion

The primary aim of this prospective study was to evaluate the feasibility of a comprehensive, multidimensional language assessment—encompassing receptive and expressive vocabulary, receptive comprehension, phonology and articulation—in children aged 4 to 6 years with ASD, a subgroup underrepresented in the literature [70]. Notably, our sample was intentionally inclusive of children with highly heterogeneous language profiles, the majority of whom were minimally or nonverbal. A secondary objective was to examine the degree of concordance between standardized language assessments and more commonly used tools in this population, namely developmental batteries and hetero-assessments.

### 4.1. Feasibility of Standardized Language Testing in Children with ASD Aged 4 to 6 Years

We employed standardized assessments aligned with French clinical guidelines for preschoolers [16,57,58], ensuring a psychometric rigor and relevance to real-world clinical practice [2]. We therefore chose EVIP, E.CO.S.SE and EVALO 2–6. Rasch modeling confirmed the appropriateness of these tools for most participants, while highlighting varying levels of accessibility depending on the specific test. However, these tools—normed on typically developing children—do not always capture the developmental specificity of ASD profiles. Indeed, the difficulty levels inherent in these assessments, which are based on chronological age norms, did not consistently reflect the actual abilities of our sample. Age-equivalent scoring, as used in some prior studies [18], would likely have reduced variability due to floor effects [71], and such methods may compromise the psychometric robustness of the instruments [72,73].

We found moderate to strong correlations among the various components of language, suggesting a coherent pattern of functionally interdependent language skills. However, strong correlation between, for example, word retrieval accuracy and speed, and phonological accuracy of the produced word suggested potential redundancy, possibly reflecting overlapping language processes (e.g., the speed of phonological representation activation and the use of articulatory codes). Although our initial aim was to conduct a broad language assessment across multiple domains—extending beyond the typical expressive/receptive dichotomy [18,44], our findings supported a more focused approach. Clinically, these findings suggest that practitioners may enhance efficiency by selecting targeted assessments instead of administering comprehensive test batteries, especially when evaluating children with limited attention spans or behavioral difficulties, like children with ASD [74]. Test selection should be strategically tailored to the child’s individual profile and the specific clinical questions, and informed by empirically derived performance patterns characteristic of this population’s atypical developmental trajectories [18].

Interestingly, children in our sample performed more poorly on the expressive lexicon test than on the receptive lexicon test—contrary to diagnostic classification specifications [1] and to findings in earlier studies, which typically report more marked deficits in receptive language [18,44]. Several factors may explain this discrepancy.

Firstly, our study included children across a wide spectrum of verbal abilities and cognitive profiles. Based on parental reports, nearly half of our sample (45%) would be considered nonverbal according to the definition proposed by Rutter and colleagues [17]. In contrast, estimates in the literature range from 50 to 74% for preschool-aged children [52]. Furthermore, in our sample, 31 children scored 0 on at least one language test and 4 children scored 0 on all tests. This pattern may be indicative of a selection bias associated with recruitment from specialized clinical services, which commonly serve children with more pronounced expressive vocabulary impairments rather than receptive vocabulary deficits. It is important to note that the term «minimally verbal» to describe children who either fail to develop functional verbal language or exhibit only minimal verbal expression is inconsistently defined across studies [22]. Some use a vocabulary threshold [75]; others apply the Autism Diagnostic Observation Schedule, second edition (ADOS-2; [76]) Module 1 criteria for children over 30 months who remain at the single-word stage [77]; and still others use hetero-assessments like the MacArthur-Bates Communicative Development Inventories (CDI; [78]) to identify children whose expressive skills do not exceed those of an 18-month-old [79]. Consequently, prevalence estimates vary widely—from 13% using CDI criteria to 28% using ADOS-2 Module 1 [22]. Few studies have specifically targeted this subgroup, underscoring the need for adapted methodologies and dedicated research [22]. Clinically, this language heterogeneity highlights the need for assessment tools that can capture the full continuum of language abilities—from nonverbal and minimally verbal to verbal profiles, particularly within this age group.

Secondly, an alternative explanation could be attributed to the nature of the tests used to assess word knowledge. More generally, our receptive and expressive assessments can both be conceptualized as recognition memory tasks employing different cues [80]. In the EVIP (receptive test), participants can benefit from both the phonological cue (word name) provided by the examiner and the visual referent among four simultaneously presented pictures. Conversely, the Denomination-Lex 1 subtest (expressive test) presents participants with only a visual referent, requiring the explicit retrieval of both phonological and articulatory representations associated with the picture. While both assessment formats provide memory cues, their cognitive demands differ substantially. This methodological distinction may provide an explanation for the observed discrepancy in performance between receptive and expressive vocabulary measures, with children with ASD aged 4 to 6 demonstrating superior performance in receptive vocabulary compared to expressive vocabulary, as evidenced in our study.

Finally, methodological differences in scoring approaches—raw scores versus Rasch scores—can substantially influence observed pattern, depending on whether children are evaluated relative to normative peers or in terms of the internal structure of their language ability. This distinction is critical: raw scores count correct responses and depend heavily on the specific set of items used, whereas Rasch scores provide objective interval-scale measurement by estimating child’s ability on a scale independent of particular items administered—making them more reliable for clinical interpretation [81].

Consequently, the discrepancy between our findings and those reported in diagnostic classification specifications [1] and other studies [18,44] is likely attributable to methodological differences. From a theoretical standpoint, our findings are consistent with Chiat’s model [82] of lexical acquisition, which explains the recognition–recall asymmetry in vocabulary development. Within this framework, fast mapping (initial phonological encoding and provisional semantic association) precedes slow mapping (phonological, semantic and articulatory consolidation in the lexicon). This developmental sequence necessarily implies that receptive lexical knowledge—reflecting successful fast mapping of phonological and semantic word codes—emerges before expressive production, which additionally requires consolidation of articulatory motor programs. This developmental principle is empirically well-established: in typically developing infants, comprehension vocabulary (around 12 months) systematically precedes production vocabulary (around 18 months) [78]. Accordingly, the pattern observed in our study (superior receptive relative to expressive lexical performance) aligns with typical developmental sequences. This suggests that, when data are collected with methodological rigor and analyzed using appropriate psychometric models, quantitative evidence may reveal a partially preserved developmental organization in children with ASD. Theoretically, this interpretation has important implications for understanding language development in ASD. Rather than indicating a fundamental deviation from typical language acquisition pathways, the receptive-expressive discrepancy identified here may reflect a similar, though delayed and potentially protracted, developmental trajectory. Clinically, this pattern suggests that intervention strategies should account for this developmental delay, prioritizing receptive vocabulary enhancement as a foundation for subsequent expressive language growth, while simultaneously addressing the phonological and articulatory retrieval difficulties that constrain expressive output.

### 4.2. Exploring the Concordance of Standardized Language Tests, Developmental Battery and Hetero-Assessments

Our findings indicate that while concordance between standardized language tests, developmental battery and hetero-assessments was occasionally observed, it was not systematic. Specifically, we found positive correlations between scores on the Receptive and Expressive language subdomains of the PEP-3 developmental battery and the Receptive and Expressive language subdomains from two hetero-assessments—the IFDC (retrospective) and VABS-II (current). However, correlations between retrospective (IFDC) and current (VABS-II) hetero-assessments were largely absent, raising concerns about the reliability and consistency of parent-reported retrospective data. Notably, both the VABS-II and PEP-3 subdomains for Receptive and Expressive language significantly correlated with several standardized test scores (covering receptive and expressive vocabulary, comprehension, phonology and articulation). These results indicate that hetero-assessments, developmental batteries and standardized tests for evaluating language performance were complementary but not interchangeable. Previous studies show that correlations between direct assessments and parent-reported measures tend to be lower for receptive language than for expressive language in children with ASD [83,84]. This finding highlights the complementary nature of these assessment approaches, emphasizing the importance of combining direct assessments and parental reports to obtain a more comprehensive profile of language abilities in children with ASD. However, in our study, the lack of correlation between retrospective IFDC scores, which depends on parental recall to characterize their child’s early development, and standardized tests revealed the limitations of relying on parental recall, which is subject to memory biases, omissions and inaccuracies. These findings carry important clinical implications for diagnostic evaluation and intervention planning. Clinicians should be aware that hetero-assessments, developmental batteries and standardized tests each yield distinct yet complementary—rather than interchangeable—perspectives on language functioning in children with ASD aged 4 to 6. Critically, current hetero-assessments provide more reliable and complementary information than retrospective measures. Consequently, combining direct assessments—both standardized tests and developmental batteries—with current hetero-assessments is essential for achieving a comprehensive and nuanced understanding of language abilities in this population.

Concerning the predictive value of developmental battery and hetero-assessments, linear regression models revealed several distinct patterns: (1) Receptive language assessed through the PEP-3 was the strongest predictor of lexical breadth (EVIP) and oral comprehension of topological terms (Understanding of topological terms subtest) in standardized tests; (2) Expressive language in the PEP-3 best predicted word retrieval accuracy and speed (Denomination-Lex 1 scoring), as well as lexical breadth and organization (Designation from a cue subtest); (3) Receptive language from the VABS-II emerged as the strongest predictor of articulatory skills (Orofacial and lingual praxis subtest), while oral sentence comprehension (E.CO.S.SE) was best predicted by a combination of PEP-3 Expressive language and VABS-II Receptive language; (4) For phonological accuracy of the produced word (Denomination-Phono 1 scoring), both Expressive language from VABS-II and Receptive language from PEP-3 were significant concurrent predictors. Importantly, none of the subdomains of the IFDC predicted performance on any standardized test, confirming its limited validity as a retrospective tool due to its reliance on parental recall to characterize child’s early development. These results support the conclusion that current parent-reported observations (e.g., VABS-II) and developmental batteries (e.g., PEP-3) were predictive of specific language abilities, but their contributions varied across domains. They did not consistently map onto receptive or expressive functions as measured by standardized tests, likely due to differences in test structure, cognitive demand and assessment context.

Our findings highlight that standardized direct assessments cannot be reduced to or replaced by global developmental evaluations or hetero-assessments. While developmental batteries like the PEP-3 offer a broader overview that may combine distinct language processes (e.g., word comprehension, sentence comprehension, instruction following), standardized tests allow for finer-grained, function-specific analysis. This distinction is critical when assessing children with ASD, who often present with atypical developmental trajectories and variable language profiles. As emphasized by Kover and colleagues [18], direct assessments provide real-time, objective observations under controlled conditions, reducing biases associated with parental reports, which may be influenced by environmental factors, subjective experiences or expectations. Likewise, Posar and Visconti [22] advocate for the systematic use of standardized tools to avoid interpretative ambiguities across studies and clinical contexts. While hetero-assessments and developmental batteries offer complementary perspectives, they must be interpreted alongside standardized measures to ensure diagnostic accuracy and appropriate intervention planning. For clinical practice, these findings suggest that clinicians should prioritize standardized direct assessments to identify specific language targets and monitor progress, while employing developmental batteries and hetero-assessments to capture how language skills translate into functional communication in everyday contexts. This multimethod approach ensures both analytical precision and ecological validity in characterizing the language abilities of children with ASD aged 4 to 6.

Additional analyses revealed that specific subtests were particularly predictive of performance on hetero-assessments and developmental battery: (1) Oral sentence comprehension (E.CO.S.SE) subtest was the best predictor of Receptive language in both the VABS-II and PEP-3; and (2) the combination of articulation skills (Orofacial and lingual praxis subtest) and word retrieval accuracy and speed (Denomination-Lex 1 scoring) best predicted Expressive language in these same tools. However, despite these predictive links, global language subdomains in the VABS-II and PEP-3 could not be fully explained by any one group of standardized tests. This finding reflects the broader issue of construct overlap in global assessments, which often conflate multiple linguistic and cognitive processes under a single score. In contrast, standardized tests target specific competencies, allowing for greater diagnostic precision.

Although preliminary, this concordance analysis suggests that relying on a single type of assessment—whether standardized, developmental or parental—risks overlooking important aspects of language function in preschoolers with ASD. For clinical practice, we recommend a multimodal assessment that integrates: (1) standardized testing to obtain fine-grained, objective measures for identifying specific intervention targets; (2) developmental batteries to provide broader functional insights into the child’s overall developmental level; and (3) current hetero-assessments to capture real-world contextual observations of communication behaviors. This integrated approach offers the most comprehensive understanding of a child’s language skills [85,86] and supports more precise, individualized intervention planning. However, this approach is time- and resource-intensive, requiring clinicians to balance its benefits against practical constraints. Its application should therefore be carefully considered in light of specific clinical goals and research priorities. Given the resource-intensive demands of comprehensive multimodal assessment, our forthcoming study [87] proposes a streamlined, clinically feasible assessment protocol specifically adapted to the heterogeneous language abilities observed in children with ASD aged 4 to 6. This protocol, grounded in data-driven identification of distinct language profiles (verbal, minimally verbal and nonverbal), seeks to preserve psychometric rigor while reducing assessment burden—addressing a critical need in clinical contexts where time and resources are limited. Future studies with larger samples are needed to refine this integrative model and determine how best to balance thoroughness with feasibility.

### 4.3. Limitations and Future Directions

Our findings should be interpreted in light of several limitations. First, despite the large number of measures collected, our sample size remained small and findings should be interpreted as preliminary. Nevertheless, this study was ambitious in its design, as it investigated a population that is rarely studied using specialized tools that are infrequently employed in research. To achieve adequate statistical power, a sample size of approximately 115 participants was estimated a priori using G*Power (version 3.1.9.7). A larger cohort would enable more sophisticated analyses such as network modeling or evaluation of hierarchical reticular models. Second, our study lacked a directly assessed comparison group, which limits interpretation of relative delays [18]. Incorporating a well-matched typically developing control group—matched for chronological age and nonverbal cognitive abilities [88]—would strengthen analyses. However, selecting appropriate control groups in ASD research is complex, as researchers must decide whether the control group should be matched for chronological age, developmental age in terms of receptive or expressive language abilities, consist of infants at low risk for ASD evaluated longitudinally [27] or include children with other neurodevelopmental disabilities [3,44,85]. Moreover, heterogeneity in ASD samples combined with homogeneity in control groups can introduce generalization biases in the interpretation of results [4]. As a result, we, like others [4,71], focused exclusively on children with ASD.

Standardized tests, while offering objectivity and psychometric reliability, also present challenges. Poor test performance may reflect difficulties beyond language ability, such as attention deficits, social interaction impairments, imitation difficulties, irregular eye contact during interaction, a particular use of language, such as echolalia, or test engagement issues [74]. Moreover, standardized assessments often rely on conformity and structured demands to ensure a valid assessment [74] that may mask the true nature of language difficulties in children with ASD [18]. Alternative paradigms designed for younger children—more accessible and less demanding than traditional tests, such as gaze-based responses (e.g., [89]), offer promising avenues but require specialized resources and lack standardized norms. Additionally, while the Rasch analysis supported our choice of tests by demonstrating interval-scale measurement objectivity and confirming test validity, opportunities for improving the test selection remain. In fact, the EVIP version used was an older paper-based edition from 1993. Since 2023, a French adaptation of the PPVT-5 [90] has become available, paired with the EVT-3 [91] for expressive vocabulary. As our protocol was finalized and initiated prior to that date, switching instruments mid-study would have compromised methodological consistency (examiner training, procurement and cross-participants comparability) and would have required protocol amendments and re-approval. However, using the PPVT-5 alongside the EVT-3 would likely have offered a more precise and balanced assessment of receptive and expressive vocabulary. Finally, EVALO 2–6 was preferred over other tests—for instance, the Battery for examining language functions in children aged 3 to 6 years old (or batterie d’EXAmen des fonctions Langagières chez l’enfant de 3 à 6 ans, EXALang 3–6; [92])—because it is relatively more recent and affords a comprehensive assessment of both receptive and expressive oral language, closely aligning with our study objectives.

All instruments and normative data employed in this study were developed and standardized in French populations. While lexical items are indeed specific to French-speaking populations, the selected instruments are widely used in research and constitute validated French translations and adaptations of established English-language assessments—specifically, the EVIP (adapted from the PPVT-R) and the E.CO.S.SE (adapted from the TROG). These tests and their associated Rasch scores provide objective measurement of universal core oral language components, including receptive and expressive vocabulary (with corresponding articulatory skills) and oral sentence comprehension.

Future research should expand sample sizes and develop streamlined, adapted protocols suitable for clinical use with preschoolers with ASD. This need is emphasized in the literature, which calls for tailored assessments to guide effective interventions [93].

## 5. Conclusions

This study demonstrated that most children with ASD aged 4 to 6 could successfully complete standardized language assessments despite their notably heterogeneous language profiles, including many minimally verbal participants. Importantly, children performed better on receptive than expressive lexicon tests, suggesting that comprehension-based assessments might better capture underlying word knowledge without confounding production difficulties. This finding challenged previous reports of more pronounced receptive language deficits and highlighted the importance of considering assessment modality when interpreting language skills in ASD. Reducing verbal demands appear to facilitate identification of underlying lexical competencies, suggesting that adapted assessment approaches could help distinguish performance limitations from genuine comprehension deficits.

Our findings also suggest that standardized tests provide more precise and detailed language profiles than developmental batteries or hetero-assessments alone. The partial but non-equivalent concordance between assessment methods underscored their complementary rather than interchangeable nature. While developmental batteries and hetero-assessments provided valuable contextual information, they could not substitute for the precision and objectivity of direct standardized testing. Given the multifaceted nature of language development in ASD [18], an integrated, multimodal assessment approach appears essential for accurately identifying both language strengths and therapeutic targets in this population.

Overall, our results support the feasibility and clinical relevance of adapting standardized language assessments for preschool-aged children with ASD. However, given the test demands intrinsic to test modalities, adapted tools are essential for distinguishing performance limitations from genuine comprehension deficits. Moreover, future research should explore alternative paradigms designed specifically for younger children—approaches that are more accessible and less demanding than traditional standardized tests. While such paradigms offered promising avenues for assessment, they required specialized resources and lacked standardized norms.

Finally, given the critical importance of early language development for later social functioning and the unique developmental trajectories in ASD, bridging clinical practice and research through comprehensive, evidence-based language assessment protocols appear crucial for optimizing intervention outcomes during this pivotal developmental period [93].

## Figures and Tables

**Table 3:** Summary of linear regression models predicting Rasch scores on language tests from hetero-assessment and developmental battery scores (R^2^ values).

	Dependent Variables	Lexicon			Receptive Comprehension		Phonology	Articulation
		Receptive Lexicon		Expressive Lexicon				
Predictor Variables		EVIP	Designation from a Cue	Denomination-Lex 1	Understanding of Topological Terms	E.CO.S.SE	Denomination-Phono 1	Orofacial and Lingual Praxis
IFDC-12 months	Comprehension							
	Production							
IFDC-18 months	Comprehension							
	Production							
IFDC-24 months	Production							
VABS-II	Receptive language					22% ** (simple effect)		31% **
	Expressive language						55% *** (simple effect)	
PEP-3	Receptive language	43% ***			20% *		55% *** (simple effect)	
	Expressive language		19% *	52% ***		60% *** (simple effect)		
Total effect (R^2^)		43% ***	19% *	52% ***	20% *	65% *** (simultaneous effect)	62% *** (simultaneous effect)	31% **

*** *p* ≤ 0.001; ** *p* ≤ 0.01; * *p* ≤ 0.05. R^2^ values represent the variance explained by the predictors; simple effects refer to individual predictors, while simultaneous effects refer to combined predictors in the model. Gray cells indicate the absence of regression model.

**Table 4:** Summary of linear regression models predicting receptive language scores from hetero-assessment and developmental battery scores using Rasch scores on receptive language tests (R^2^ values).

		Dependent Variables	IFDC-12 Months	IFDC-18 Months	VABS-II	PEP-3
Predictor Variables			Comprehension	Comprehension	Receptive Language	Receptive Language
Lexicon	Receptive lexicon	EVIP				
		Designation from a cue				
Receptive comprehension		Understanding of topological terms				
		E.CO.S.SE			19% **	53% ***
Total effect (R^2^)					19% **	53% ***

*** *p* ≤ 0.001; ** *p* ≤ 0.01. R^2^ values represent the variance explained by the predictors; simple effects refer to individual predictors, while simultaneous effects refer to combined predictors in the model. Gray cells indicate the absence of a regression model.

**Table 5:** Summary of linear regression models predicting expressive language scores from hetero-assessment and developmental battery scores using Rasch scores on expressive language tests (R^2^ values).

		Dependent Variables	IFDC-12 Months	IFDC-18 Months	IFDC-24 Months	VABS-II	PEP-3
Predictor Variables			Production	Production	Production	Expressive Language	Expressive Language
Lexicon	Expressive lexicon	Denomination-Lex 1				57% *** (simple effect)	52% *** (simple effect)
Phonology		Denomination-Phono 1					
Articulation		Orofacial and lingual praxis				39% *** (simple effect)	24% *** (simple effect)
Total effect (R^2^)						66% *** (simultaneous effect)	60% *** (simultaneous effect)

*** *p* ≤ 0.001. R^2^ values represent the variance explained by the predictors; simple effects refer to individual predictors, while simultaneous effects refer to combined predictors in the model. Gray cells indicate the absence of a regression model.

## Data Availability

All data generated or analyzed during this study are included in this published article and its Supplementary Information Files.

## Supplementary Materials

The following supporting information can be downloaded at: https://www.mdpi.com/article/10.3390/children12121596/s1, Supplementary File S1: Table S1: Descriptive characteristics of the participants. Reference [94] is cited in the supplementary materials. Supplementary File S2: Figure S1: Wright map of the Denomination-Lex 1 scoring; Figure S2: Wright map of the Denomination-Lex 2 scoring; Figure S3: Wright map of the Designation from a cue subtest; Figure S4: Wright map of the Understanding of topological terms subtest; Figure S5: Wright map of the E.CO.S.SE; Figure S6: Wright map of the Denomination-Phono 1 scoring; Figure S7: Wright map of the Denomination-Phono 2 scoring. Figure S8: Wright map of the subtest Orofacial and lingual praxis. Supplementary File S3: Table S2: Inter-correlation matrix of Rasch scores on language tests; Table S3: Inter-correlation matrix between scores on hetero-assessments and scores on developmental battery; Table S4: Inter-correlation matrix between Rasch scores on language tests and scores on hetero-assessments; Table S5: Inter-correlation matrix between Rasch scores on language tests and scores on the developmental battery. Supplementary File S4: Supplementary stepwise regression analysis.
