# Supplementary material for: Assessing Language Skills in Children Aged 4 to 6 Years with Autism Spectrum Disorder: A Prospective Study
Source: Children (Basel). 2025 Nov 24;12(12):1596. doi: 10.3390/children12121596 (PMC12732180; doi:10.3390/children12121596)
Supplement: Supplementary file 1 [file children-12-01596-s001.zip › Supplementary File S1.pdf]

Assessing Language Skills in Children Aged 4 to 6 Years with Autism Spectrum Disorder: A Prospective Study

Supplementary file S1

Table S1 Descriptive characteristics of the participants

**Measure**	**Mean**	**SD**	**Range**
**Chronological age (months)**	63.50	8.25	48-82
**VABS-II-adaptive behavior composite score (*N* = 44)**	41.3	19.3	20-98
- VABS-II-communication domain standard score	- 48.0	- 18.0	- 20-93
- VABS-II-receptive language v-scale score	- 7.52	- 3.67	- 1-14
- VABS-II-expressive language v-scale score	- 3.45	- 3.35	- 1-14
- VABS-II-written language v-scale score	- 11.1	- 3.12	- 3-20
- VABS-II-daily living skills domain standard score	- 59.0	- 22.2	- 20-119
- VABS-II-personal autonomy v-scale score	- 4.77	- 3.92	- 1-16
- VABS-II-domestic autonomy v-scale score	- 12.6	- 4.10	- 4-24
- VABS-II-community life v-scale score	- 9.70	- 3.16	- 1-17
- VABS-II-socialization domain standard score	- 51.1	- 20.7	- 20-96
- VABS-II-interpersonal relationships v-scale score	- 7.11	- 3.51	- 1-15
- VABS-II-play and free time v-scale score	- 7.07	- 4.30	- 1-15
- VABS-II-adaptation v-scale score	- 7.89	- 3.13	- 1-13
- VABS-II-motor skills domain standard score	- 43.8	- 22.4	- 20-89
- VABS-II-gross motor v-scale score	- 7.27	- 5.07	- 1-19
- VABS-II-fine motor v-scale score	- 5.09	- 4.04	- 1-13
**PEP-3 (*N* = 33)**			
- PEP-3-communication developmental age (*N* = 32)	- 26.5	- 10.5	- 11.0-56.7
- PEP-3-preverbal/verbal cognition developmental age	- 34.7	- 14.7	- 13-78
- PEP-3-expressive language developmental age	- 21.5	- 9.36	- 11-46
- PEP-3-receptive language developmental age	- 21.6	- 10.5	- 11-52
- PEP-3-motricity developmental age (*N* = 32)	- 31.6	- 7.11	- 14.7-42.3
- PEP-3-fine motor developmental age	- 34.0	- 10.3	- 11-51
- PEP-3-gross motor developmental age	- 30.0	- 6.67	- 11-38
- PEP-3-oculomotor imitation developmental age	- 30.1	- 6.90	- 13-42
**Age of first words (months) (*N* = 45)**	23.3	14.8	6-60
**Age of first sentences (months) (*N* = 25)**	51.5	12.1	24-72
**Professions and socio-professional categories [1] of parents**	***N* = 92 excluding 2 non-applicable data (percentage of the sample)**		
1. Farmer	0		
2. Craftsman/trader	5 (5%)		
3. Higher intellectual profession	2 (2%)		
4. Intermediate profession	10 (11%)		
5. Employee	28 (30%)		
6. Worker	7 (8%)		
7. Retired	0		
8. Inactive	40 (44%)		
- Including mother	30 (75%)		
- Including father	10 (25%)		
- Including both parents	5 (5%)		

Standard Deviation (SD)

When data are missing, the number of participants (*N*) is adjusted accordingly.

[1] According to the professions and socio-professional categories nomenclature of the National Institute of Statistics and Economic Studies [1].

Vineland Adaptative Behaviour Scales, second edition (VABS-II): the mean of the adaptive behavior composite score and of the domain standard scores is 100, with a SD of 15. The mean of the v-scale scores is 15, with a SD of 3.
Psychoeducational Profile, third edition (PEP-3): the developmental ages are expressed in months.

Reference

1. Professions and socio-professional categories 2003. Available online: https://www.insee.fr/fr/metadonnees/pcs2003/categorieSocioprofessionnelleAgregee/1?champRecherche=true (accessed on 24 September 2024).
