# Supplementary material for: Assessing Language Skills in Children Aged 4 to 6 Years with Autism Spectrum Disorder: A Prospective Study
Source: Children (Basel). 2025 Nov 24;12(12):1596. doi: 10.3390/children12121596 (PMC12732180; doi:10.3390/children12121596)
Supplement: Supplementary file 1 [file children-12-01596-s001.zip › Supplementary File S2.pdf]

Assessing Language Skills in Children Aged 4 to 6 Years with Autism Spectrum Disorder: A Prospective Study

Supplementary file S2

Item difficulty analysis (1-parameter Rasch model) of receptive vocabulary

Rasch analysis of lexical breadth and organization (Designation from a cue subtest):

The person reliability value was relatively high (.796, $p < .001$), indicating that the test reliably measured its intended construct. Item infits predominantly fell within the acceptable reference interval [0.7;1.3], with only 2 items < 0.7 and 1 item > 1.3, suggesting adequate model fit. As illustrated in Figure S1, there was minimal overlap between participant and item distributions: 15 of the 41 participants scored 0 and all 22 items were successfully completed by at least one participant.

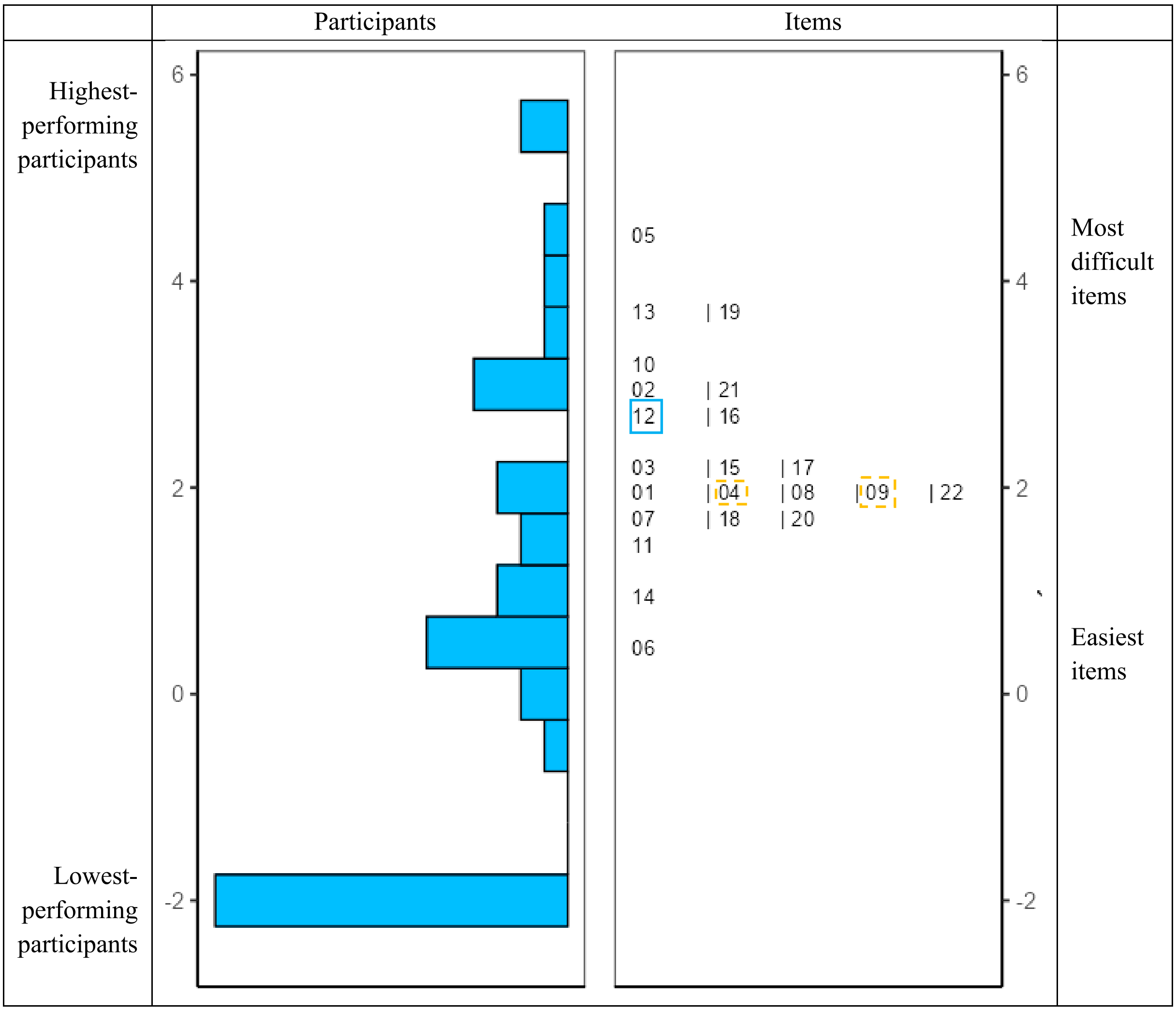

Figure S1 Wright map of the Designation from a cue subtest
Orange (dashed line): item infits < 0.7; blue (solid line): item infits > 1.3

Excluding the 15 participants who scored 0, the receptive vocabulary distribution function became more uniform, indicating that the majority of the sample demonstrated varied response patterns across items. The Rasch analysis confirmed that the Designation from a cue subtest was appropriate for 63% of participants who provided differentiated responses across items. This assessment proved suitable for evaluating lexical comprehension in children with ASD, even within a sample characterized by highly heterogeneous language abilities. However, for the remaining 37%, the test proved to be too challenging and exceeded their current ability level (lowest negative score on the Rasch scale).

Additionally, the Rasch analysis revealed that items at equivalent difficulty levels demonstrated comparable difficulty within our sample. Consequently, the assessment could be abbreviated by selecting representative items from each difficulty level. Finally, item difficulty rankings did not necessarily correspond to the skill hierarchy observed in our atypical sample, suggesting that typical developmental progressions may not align with the unique developmental trajectories characteristic of this population.

Item difficulty analysis (1-parameter Rasch model) of expressive vocabulary

Rasch analysis of word retrieval accuracy and speed (Denomination-Lexicon scoring)

To maintain consistency with the Rasch dichotomous model, and given that the polytomous Rasch model (scores of 0, 1 and 2) was not feasible due to insufficient sample size, we dichotomized the scoring system into two components: Denomination-Lex 1 (1: words produced on initial attempt, 0: failure) and Denomination-Lex 2 (1: words produced on initial attempt or following phonemic cueing, 0: failure). This approach enabled differentiation between participants who benefited from phonemic assistance versus those who did not. For the present analyses, we focused exclusively on Denomination-Lex 1 to assess purely the efficiency and accuracy of lexical retrieval in response to visual stimuli, without any external facilitation.

Denomination-Lex 1

The person reliability value was relatively high (.700, $p < .001$), indicating that the test reliably measured its intended construct. Item infits predominantly fell within the acceptable reference interval [0.7;1.3], with only 4 items < 0.7 and 3 items > 1.3, suggesting adequate model fit. As shown in Figure S2, there was minimal overlap between participant and item distributions: 22 of the 42 participants scored 0 and 3 of the 40 items out were failed by all participants (these items were excluded from the Rasch analyses).

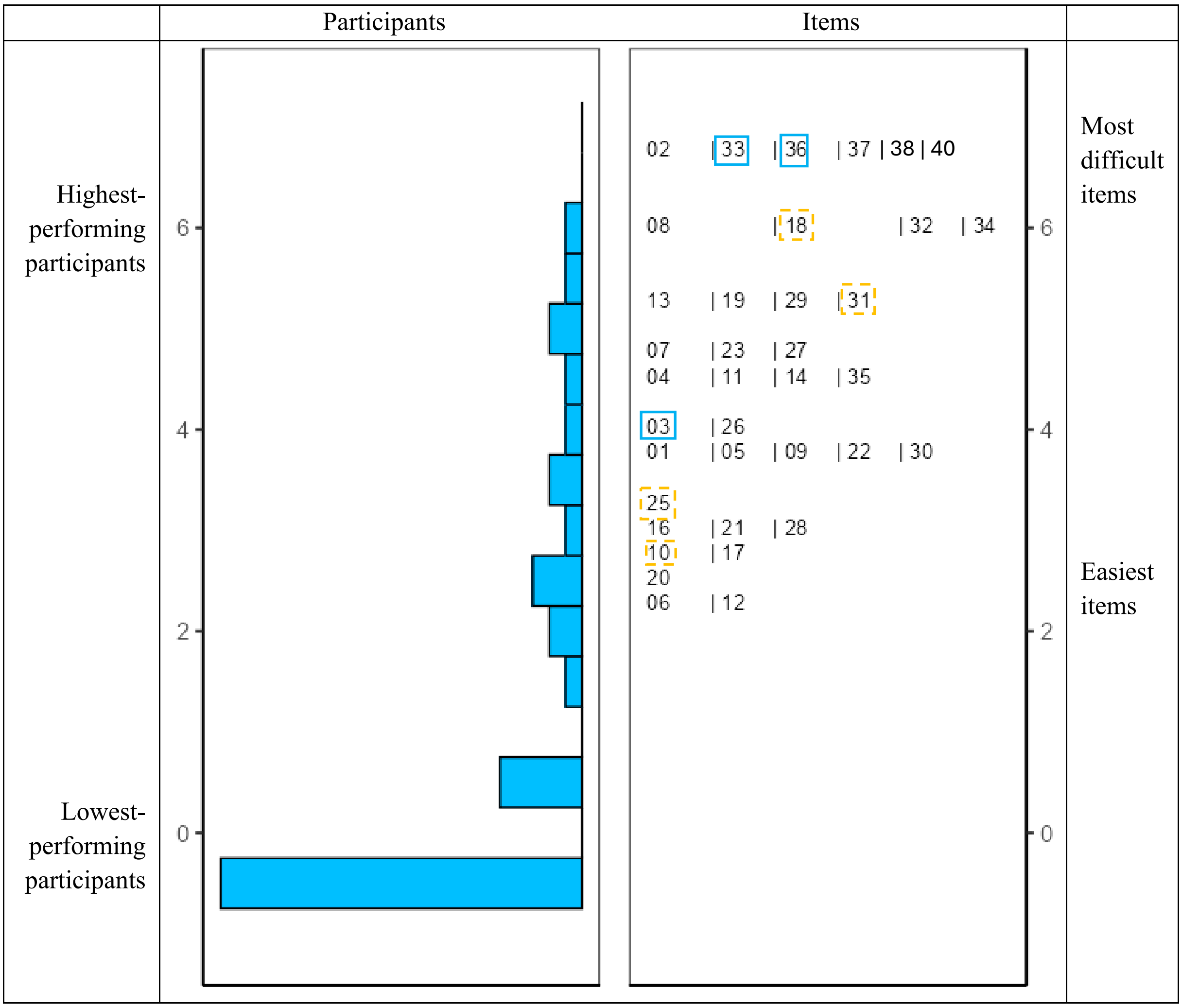

Figure S2 Wright map of the Denomination-Lex 1 scoring
Orange (dashed line): item infits < 0.7; blue (solid line): item infits > 1.3

Excluding the 22 participants who scored 0, the expressive vocabulary distribution function became globally uniform, indicating that the minority of the sample demonstrated varied response patterns across items. The Rasch analysis confirmed that the Denomination-Lex 1 subtest was appropriate for 48% of the participants, who provided differentiated responses across items. This assessment proved suitable for evaluating lexical access quality and speed in children with ASD, even within a sample characterized by highly heterogeneous language abilities. However, for the remaining 52%, the test proved to be too challenging and exceeded their current ability level (lowest negative score on the Rasch scale).

Additionally, the Rasch analysis revealed that items at equivalent difficulty levels demonstrated comparable difficulty within our sample. Consequently, the assessment could be abbreviated by selecting representative items from each difficulty level. Finally, item difficulty rankings did not necessarily correspond to the skill hierarchy observed in our atypical sample, suggesting that typical developmental progressions may not align with the unique developmental trajectories characteristic of this population.

Denomination-Lex 2

The person reliability value was relatively high (.764, $p < .001$), indicating that the test reliably measured its intended construct. Item infits predominantly fell within the acceptable reference interval [0.7;1.3], with only 4 items < 0.7 and 1 item > 1.3, suggesting adequate model fit. As shown in Figure S3, there was minimal overlap between participant and item distributions: 20 of the 42 participants scored 0 and all 40 items were successfully completed by at least one participant.

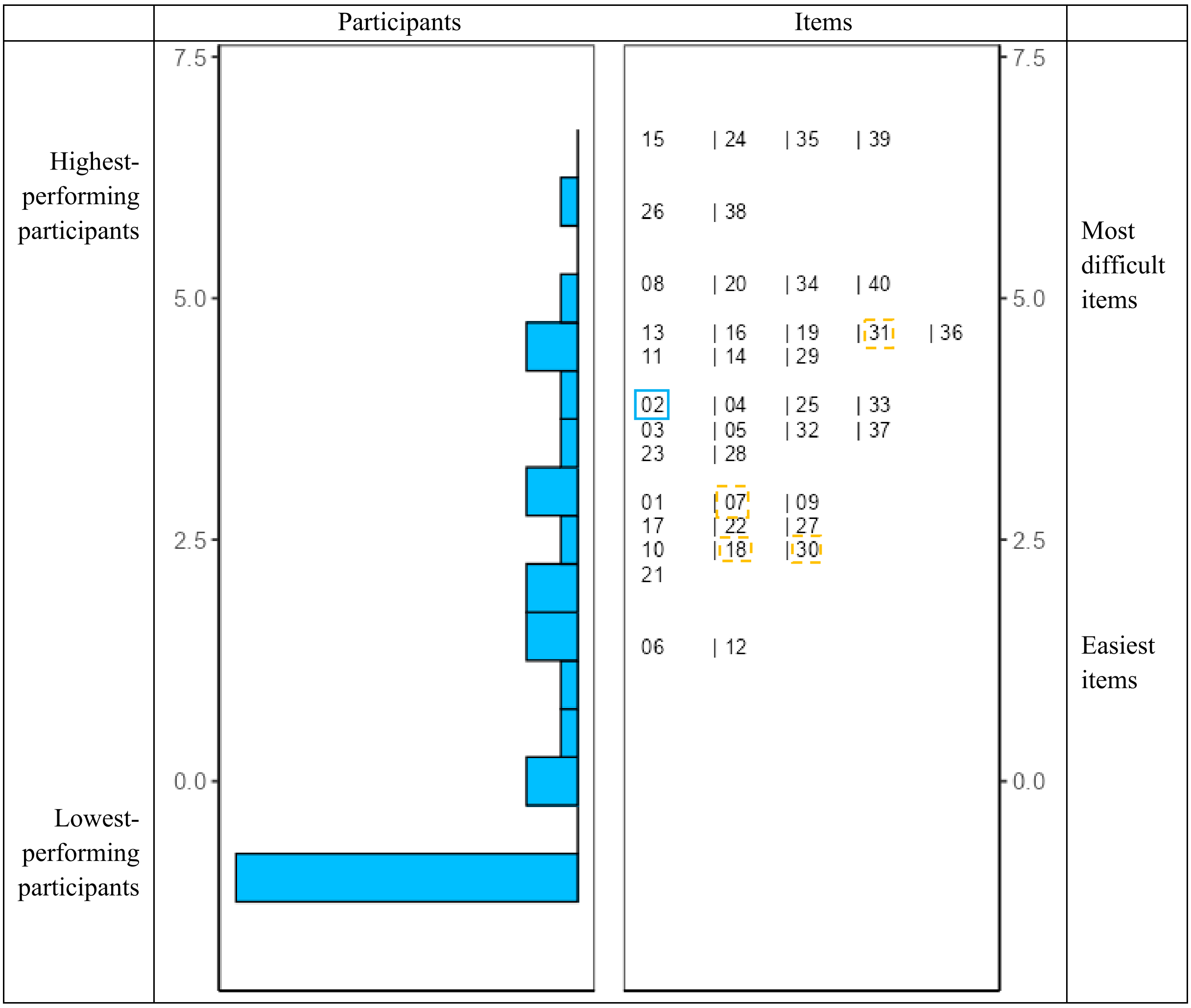

Figure S3 Wright map of the Denomination-Lex 2 scoring
Orange (dashed line): item infits < 0.7; blue (solid line): item infits > 1.3

Excluding the 20 participants who scored 0, the expressive vocabulary distribution function became globally uniform, indicating that the majority of the sample demonstrated varied responses patterns across items. The Rasch analysis confirmed that the Denomination-Lex 2 subtest was appropriate for 52% of the participants, who provided differentiated responses across items. This assessment proved suitable for evaluating lexical access quality and speed less accurately in children with ASD, even within a sample characterized by highly heterogeneous language abilities. However, for the remaining 48%, the test proved to be too challenging and exceeded their current ability level (lowest negative score on the Rasch scale). Phonemic cueing (providing the initial phoneme) enabled successful naming of all items but facilitated improved performance in only 2 participants.

Additionally, the Rasch analysis revealed that items at equivalent difficulty levels demonstrated comparable difficulty within our sample. Consequently, the assessment could be abbreviated by selecting representative items from each difficulty level. Finally, item difficulty rankings did not necessarily correspond to the skill hierarchy observed in our atypical sample, suggesting that typical developmental progressions may not align with the unique developmental trajectories characteristic of this population.

Item difficulty analysis (1-parameter Rasch model) of oral comprehension

<u>Rasch analysis of oral comprehension of topological representation vocabulary</u> (Understanding of topological terms subtest)

The person reliability value was relatively high (.619, $p < .001$), indicating that the test reliably measured its intended construct. Item infits predominantly fell within the acceptable reference interval [0.7;1.3], with 5 items < 0.7, suggesting adequate model fit. As shown in Figure S4, there was minimal overlap between participant and item distributions: 14 of the 41 participants scored 0 and all 9 items were successfully completed by at least one participant (scores of 0 were assigned for the final 3 items to the 3 youngest children who completed only the first 6 of 9 items).

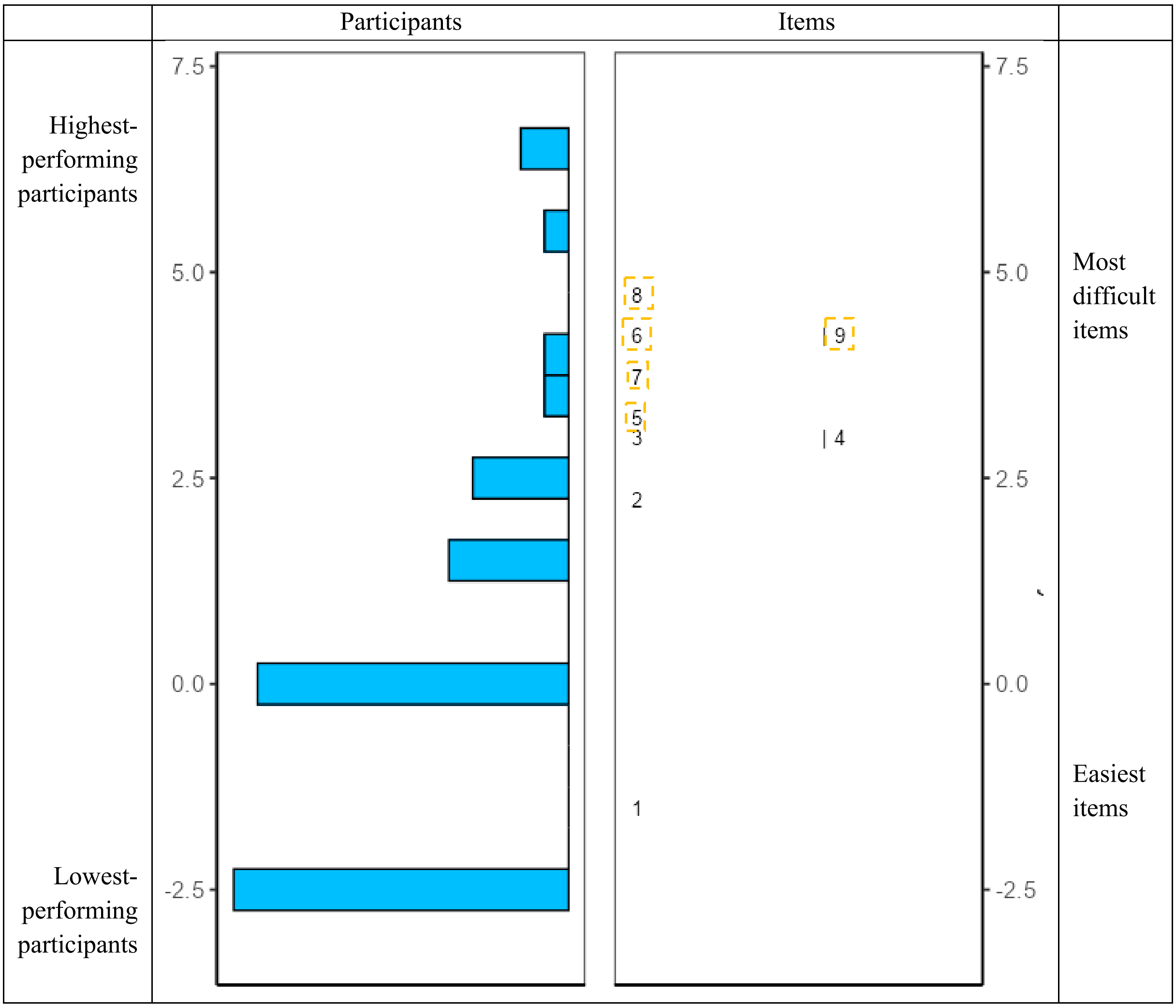

Figure S4 Wright map of the Understanding of topological terms subtest
Orange (dashed line): item infits < 0.7

Excluding the 14 participants who scored 0, the comprehension distribution function became positively skewed, indicating that the majority of the sample demonstrated varied response patterns across items. The Rasch analysis confirmed that the Understanding of topological terms subtest was appropriate for 66% of participants, who provided differentiated responses across items. This assessment proved suitable for evaluating oral comprehension of topological representation vocabulary in children with ASD, even within a sample characterized by highly heterogeneous language abilities. However, for the remaining 34%, the test proved to be too challenging and exceeded their current ability level (lowest negative score on the Rasch scale). Additionally, the Rasch analysis revealed that items at equivalent difficulty levels demonstrated comparable difficulty within our sample. Consequently, the assessment could be abbreviated

by selecting representative items from each difficulty level. Finally, item difficulty rankings did not necessarily correspond to the skill hierarchy observed in our atypical sample, suggesting that typical developmental progressions may not align with the unique developmental trajectories characteristic of this population.

Rasch analysis of oral sentence comprehension (syntaxico-semantic comprehension test – or Epreuve de COmpréhension Syntaxico-SEmantique, E.CO.S.SE –)

The person reliability value was high (.915, $p < .001$), indicating that the test reliably measured its intended construct. Item infits predominantly fell within the acceptable reference interval [0.7;1.3], with 27 items < 0.7 and 6 items > 1.3, suggesting adequate model fit. As shown in Figure S5, there was minimal overlap between participant and item distributions: 12 of the 46 participants scored 0 and 19 of the 92 items were failed by all participants (these items were excluded from the Rasch analyses).

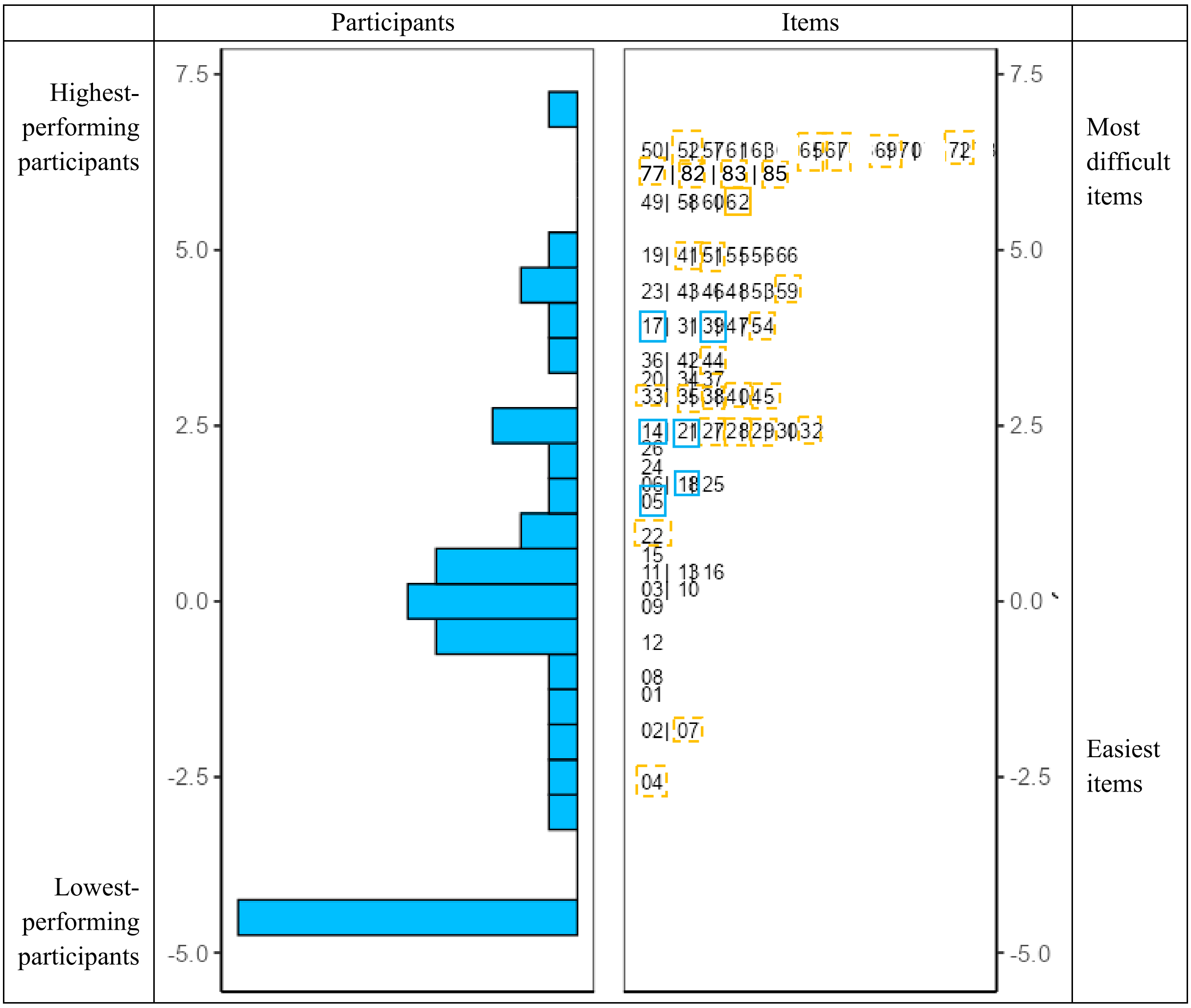

Figure S5 Wright map of the E.CO.S.SE
Orange (dashed line): item infits < 0.7; blue (solid line): item infits > 1.3

Excluding the 12 participants who scored 0, the oral comprehension distribution function approximated normality with a positive skew, indicating that the majority of the sample demonstrated varied response patterns across items. The Rasch analysis confirmed that the E.CO.S.SE was appropriate for 74% of the participants, who provided differentiated responses across items. This assessment proved suitable for evaluating oral sentence comprehension in children with ASD, even within a sample characterized by highly heterogeneous language abilities. However, for the remaining 26%, the test proved to be too challenging and exceeded their current ability level (lowest negative score on the Rasch scale).

Additionally, the Rasch analysis revealed that items at equivalent difficulty levels demonstrated comparable difficulty within our sample. Consequently, the assessment could be abbreviated by selecting representative items from each difficulty level. Finally, item difficulty rankings, as determined by chronological age norms, did not necessarily correspond to the skill hierarchy observed in our atypical sample, suggesting that typical developmental progressions may not align with the unique developmental trajectories characteristic of this population.

Item difficulty analysis (1-parameter Rasch model) of phonological quality

Rasch analysis of phonological accuracy of the produced word (Denomination-Phonology scoring)

To maintain consistency with the Rasch dichotomous model, and given that the polytomous Rasch model (scores of 0, 1 and 2) was not feasible due to insufficient sample size, we dichotomized the scoring system into two components: Denomination-Phono 1 (1: words produced on initial attempt or following phonemic cueing, 0: failure) and Denomination-Phono 2 (1: words produced on initial attempt, following phonemic cueing or after repetition modeling, 0: failure). This approach enabled differentiation between participants who benefited from repetition modeling versus those who did not. For the present analyses, we focused exclusively on Denomination-Phono 1 to assess purely the accuracy of phonological word representations in response to visual stimuli or phonemic cues, without repetition assistance, thereby ensuring lexical access to word meaning.

Denomination-Phono 1

The person reliability value was relatively high (.733, $p < .001$), indicating that the test reliably measured its intended construct. Item infits predominantly fell within the acceptable reference interval [0.7;1.3], with only 4 items < 0.7 and 1 item > 1.3, suggesting adequate model fit. As shown in Figure S6, there was minimal overlap between participant and item distributions:

21 of the 42 participants scored 0 and 1 of the 40 items was failed by all participants (this item was excluded from the Rasch analyses).

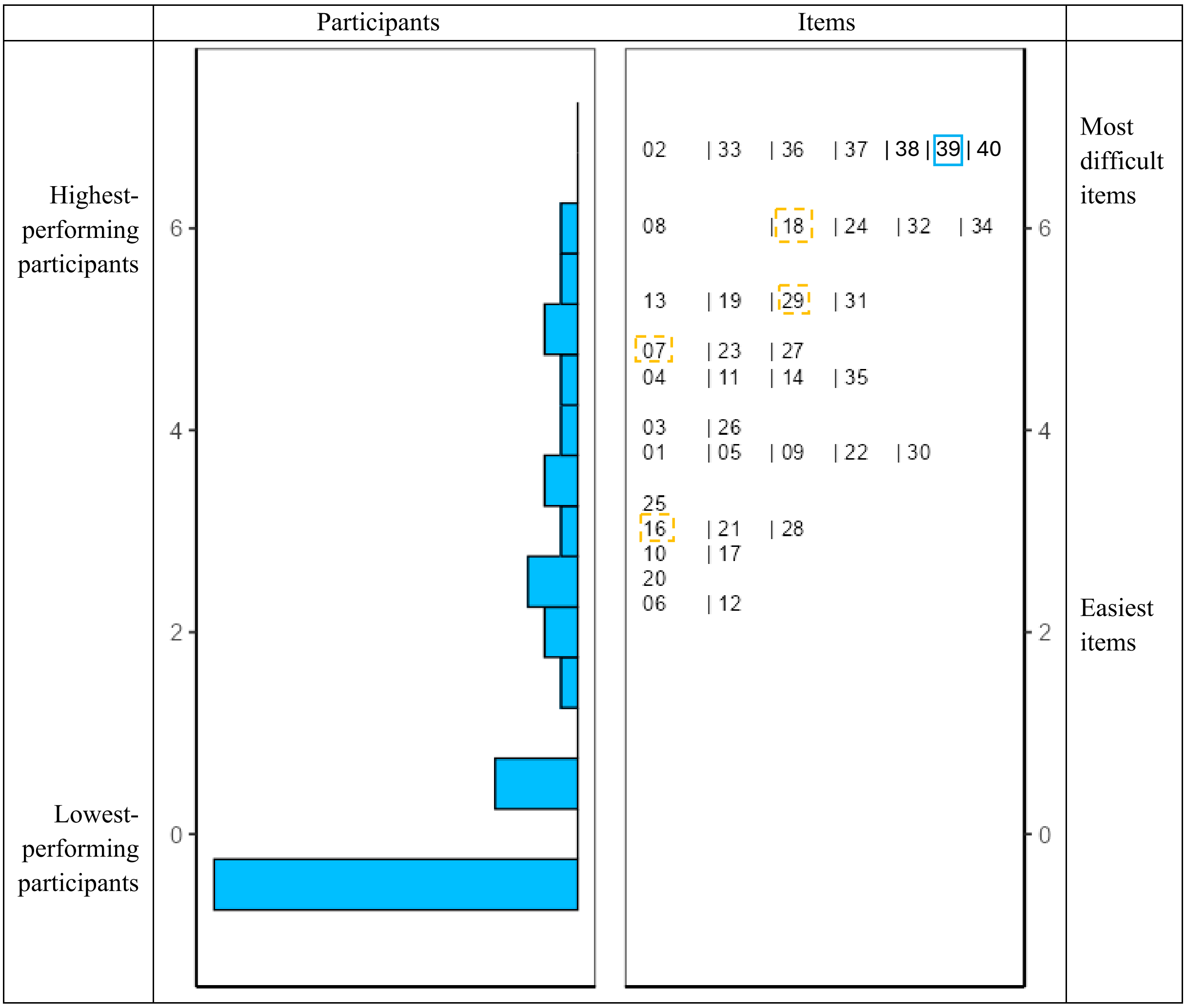

Figure S6 Wright map of the Denomination-Phono 1 scoring
Orange (dashed line): item infits < 0.7; blue (solid line): item infits > 1.3

Excluding the 21 participants who scored 0, the phonology distribution function became globally uniform, indicating that half of the sample demonstrated varied response patterns across items. The Rasch analysis confirmed that the Denomination-Phono 1 subtest was appropriate for 50% of the participants, who provided differentiated responses across items. This assessment proved suitable for evaluating the quality of phonological word representations in children with ASD, even within a sample characterized by highly heterogeneous language

abilities. However, for the remaining 50%, the test proved to be too challenging and exceeded their current ability level (lowest negative score on the Rasch scale).

Additionally, the Rasch analysis revealed that items at equivalent difficulty levels demonstrated comparable difficulty within our sample. Consequently, the assessment could be abbreviated by selecting representative items from each difficulty level. Finally, item difficulty rankings did not necessarily correspond to the skill hierarchy observed in our atypical sample, suggesting that typical developmental progressions may not align with the unique developmental trajectories characteristic of this population.

Denomination-Phono 2

The person reliability value was high (.906, $p < .001$), indicating that the test reliably measured its intended construct. Item infits predominantly fell within the acceptable reference interval [0.7;1.3], with 12 items < 0.7 and 6 items > 1.3, suggesting adequate model fit. As shown in Figure S7, there was minimal overlap between participant and item distributions: 16 of the 42 participants scored 0 and all 40 items were successfully completed by at least one participant.

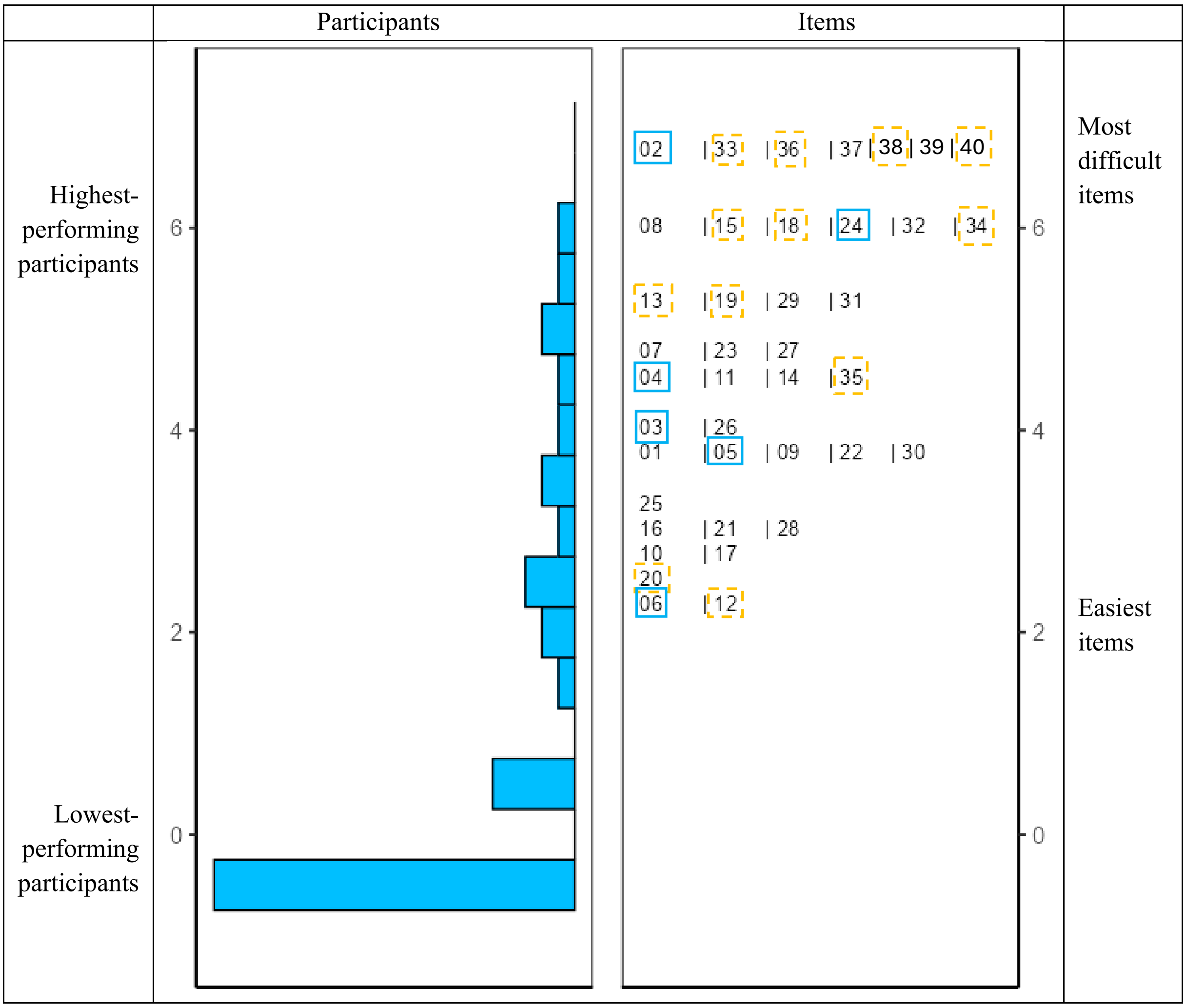

Figure S7 Wright map of the Denomination-Phono 2 scoring
Orange (dashed line): item infits < 0.7; blue (solid line): item infits > 1.3

Excluding the 16 participants who scored 0, the phonology distribution function became globally uniform, indicating that the majority of the sample demonstrated varied responses patterns across items. The Rasch analysis confirmed that the Denomination-Phono 2 subtest was appropriate for 62% of the participants, who provided differentiated responses across items. This assessment proved suitable for evaluating phonological repertoire and pronunciation quality in children with ASD, even within a sample characterized by highly heterogeneous language abilities. However, for the remaining 38%, the test proved to be too challenging and exceeded their current ability level (lowest negative score on the Rasch scale). Phonemic cueing or repetition modeling enabled successful completion of all items but yielded improved performance in only 5 participants.

Additionally, the Rasch analysis revealed that items at equivalent difficulty levels demonstrated comparable difficulty within our sample. Consequently, the assessment could be abbreviated by selecting representative items from each difficulty level. Finally, item difficulty rankings did not necessarily correspond to the skill hierarchy observed in our atypical sample, suggesting that typical developmental progressions may not align with the unique developmental trajectories characteristic of this population.

Item difficulty analysis (1-parameter Rasch model) of articulation skills

Rasch analysis of articulation skills (Orofacial and lingual praxis subtest)

The person reliability value was relatively high (.816, $p < .001$), indicating that the test reliably measured its intended construct. Item infits predominantly fell within the acceptable reference interval [0.7;1.3], with only 2 items < 0.7, suggesting adequate model fit. As shown in Figure S8, there was minimal overlap between participant and item distributions: 10 of the 41 participants scored 0 and all 18 items were successfully completed by at least one participant.

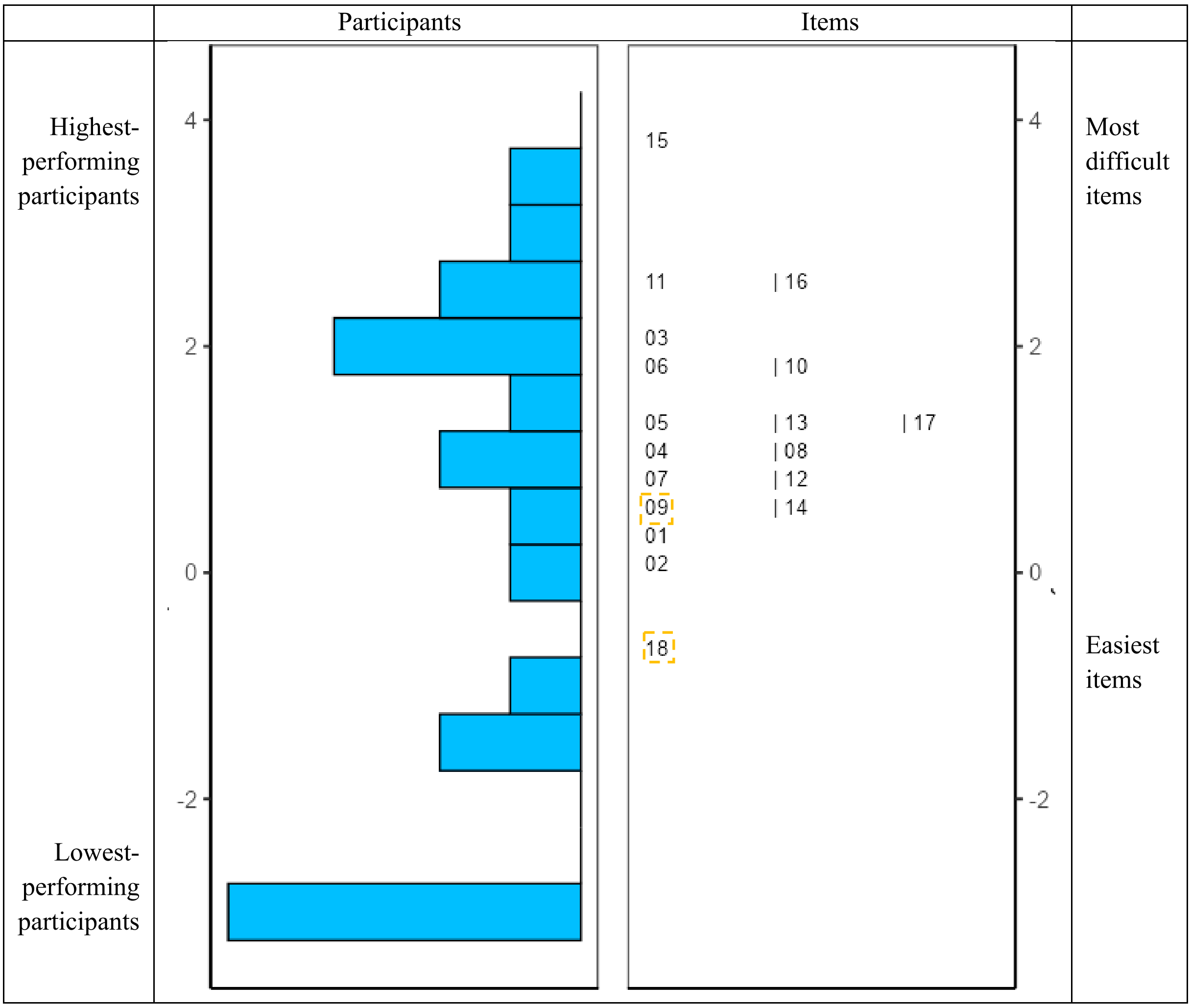

Figure S8 Wright map of the Orofacial and lingual praxis subtest
Orange (dashed line): item infits < 0.7

Excluding the 10 participants who scored 0, the articulation distribution function approximated normality with a slight negative skew, indicating that the majority of the sample demonstrated varied response patterns across items. The Rasch analysis confirmed that the Orofacial and lingual praxis subtest was appropriate for 76% of the participants, who provided differentiated responses across items. This assessment proved suitable for evaluating articulation skills in children with ASD, even within a sample characterized by highly heterogeneous language abilities. However, for the remaining 24%, the test proved to be too challenging and exceeded their current ability level (lowest negative score on the Rasch scale).

Additionally, the Rasch analysis revealed that items at equivalent difficulty levels demonstrated comparable difficulty within our sample. Consequently, the assessment could be abbreviated

by selecting representative items from each difficulty level. Finally, item difficulty rankings did not necessarily correspond to the skill hierarchy observed in our atypical sample, suggesting that typical developmental progressions may not align with the unique developmental trajectories characteristic of this population.
