# Supplementary material for: Assessing Language Skills in Children Aged 4 to 6 Years with Autism Spectrum Disorder: A Prospective Study
Source: Children (Basel). 2025 Nov 24;12(12):1596. doi: 10.3390/children12121596 (PMC12732180; doi:10.3390/children12121596)
Supplement: Supplementary file 1 [file children-12-01596-s001.zip › Supplementary File S3.pdf]

Supplementary file S3

Inter-correlation matrix of Rasch scores on language tests

Table S2 Inter-correlation matrix of Rasch scores on language tests

			Lexicon			Receptive comprehension		Phonology	Articulation
			Receptive lexicon		Expressive lexicon				
			EVIP	Designation from a cue	Denomination-Lex 1	Understanding of topological terms	E.CO.S.SE	Denomination-Phono 1	Orofacial and lingual praxis
Lexicon	Receptive lexicon	EVIP		.791***	.699***	.599***	.849***	.773***	.624***
		Designation from a cue			.733***	.619***	.752***	.813***	.537***
	Expressive lexicon	Denomination-Lex 1				.602***	.684***	.911***	.450**
Receptive comprehension		Understanding of topological terms					.613***	.632***	.477**
		E.CO.S.SE						.774***	.714***
Phonology		Denomination-Phono 1							.526***
Articulation		Orofacial and lingual praxis							

Correlations correspond to Pearson's r coefficients (***$p \leq .001$; **$p \leq .01$; *$p \leq .05$)
Peabody Picture Vocabulary Test, or Echelle de Vocabulaire en Images Peabody (EVIP)
Syntaxico-semantic comprehension test, or Epreuve de COmpréhension Syntaxico-SEmantique (E.CO.S.SE)

Inter-correlation matrix between hetero-assessment and developmental battery scores

Table S3 Inter-correlation matrix between hetero-assessment and developmental battery scores

		PEP-3		VABS-II	
		Receptive language	Expressive language	Receptive language	Expressive language
IFDC-12 months	Comprehension	.120	.237	.262	.223
	Production	.384*	.357*	.050	.100
IFDC-18 months	Comprehension	.320	.412*	.397**	.228
	Production	.449**	.415*	.225	.130
IFDC-24 months	Production	.483**	.446**	.217	.109
VABS-II	Receptive language	.554***	.436*		
	Expressive language	.586***	.684***		

Correlations correspond to Pearson's r coefficients (***$p \leq .001$; **$p \leq .01$; *$p \leq .05$)
Psychoeducational Profile, third edition (PEP-3)
Vineland Adaptative Behaviour Scales, second edition (VABS-II)
French inventories of communicative development, or Inventaires Français du Développement Communicatif (IFDC)

Inter-correlation matrix between Rasch scores on language tests and hetero-assessment scores

Table S4 Inter-correlation matrix between Rasch scores on language tests and hetero-assessment scores

		Lexicon			Receptive comprehension		Phonology	Articulation
		Receptive lexicon		Expressive lexicon				
		EVIP	Designation from a cue	Denomination-Lex 1	Understanding of topological terms	E.CO.S.SE	Denomination-Phono 1	Orofacial and lingual praxis
IFDC-12 months	Comprehension	.087	.028	.080	.018	.025	.141	.183
	Production	.028	-.062	.042	.047	.065	.029	.138
IFDC-18 months	Comprehension	.202	.030	.075	.063	.155	.130	.176
	Production	.005	-.044	.092	.054	.064	.079	.147
IFDC-24 months	Production	.140	.064	.083	.051	.215	.205	.203
VABS-II	Receptive language	.404**	.352*	.344*	.220	.464**	.395*	.542***
	Expressive language	.635***	.638***	.753***	.541***	.653***	.738***	.628***

Correlations correspond to Pearson's r coefficients (***$p \leq .001$; **$p \leq .01$; *$p \leq .05$)

Inter-correlation matrix between Rasch scores on language tests and developmental battery scores

Table S5 Inter-correlation matrix between Rasch scores on language tests and developmental battery scores

		Lexicon			Receptive comprehension		Phonology	Articulation
		Receptive lexicon		Expressive lexicon				
		EVIP	Designation from a cue	Denomination-Lex 1	Understanding of topological terms	E.CO.S.SE	Denomination-Phono 1	Orofacial and lingual praxis
PEP-3	Receptive language	.663***	.414*	.700***	.457*	.751***	.743***	.504**
	Expressive language	.561***	.386*	.709***	.365	.773***	.688***	.466*

Correlations correspond to Pearson's r coefficients (****p* ≤ .001; ***p* ≤ .01; **p* ≤ .05)
