# Supplementary material for: Assessing Language Skills in Children Aged 4 to 6 Years with Autism Spectrum Disorder: A Prospective Study
Source: Children (Basel). 2025 Nov 24;12(12):1596. doi: 10.3390/children12121596 (PMC12732180; doi:10.3390/children12121596)
Supplement: Supplementary file 1 [file children-12-01596-s001.zip › Supplementary File S4.pdf]

Assessing Language Skills in Children Aged 4 to 6 Years with Autism Spectrum Disorder: A Prospective Study

Supplementary file S4

Stepwise linear regression analyses

The results presented below focus on question (a): Whether hetero-assessments and developmental battery predict performance on standardized language tests. Here, Rasch scores from standardized language assessments were included as dependent variables, while hetero-assessment and developmental battery scores served as predictors.

Stepwise linear regression to explain receptive language scores from hetero-assessments and developmental battery using Rasch scores from receptive language standardized tests

Peabody Picture Vocabulary Test, or Echelle de Vocabulaire en Images Peabody (EVIP): one-step regression model significantly predicted the scores of the EVIP, $F(1,31) = 23.291$, $p < .001$. One predictor (Psychoeducational Profile, third edition – PEP-3 –-Receptive language) accounted for 43% of the variance in the scores of the EVIP.

Denomination-Lex 1 subtest: one-step regression model significantly predicted the scores of the Denomination-Lex 1 subtest, $F(1,27) = 29.422$, $p < .001$. One predictor (PEP-3-Expressive language) accounted for 52% of the variance in the scores of the Denomination-Lex 1.

Designation from a cue subtest: one-step regression model significantly predicted the scores of the Designation from a cue subtest, $F(1,27) = 6.415$, $p < .05$. One predictor (PEP-3-Expressive language) accounted for 19% of the variance in the scores of the Designation from a cue subtest.

Understanding of topological terms subtest: one-step regression model significantly predicted the scores of the Understanding of topological terms subtest, $F(1,27) = 6.604$, $p < .05$. One

predictor (PEP-3-Receptive language) accounted for 20% of the variance in the scores of the Understanding of topological terms subtest.

Syntaxico-semantic comprehension test, or Epreuve de COmpréhension Syntaxico-SEmantique (E.CO.S.SE): two-step regression model significantly predicted the scores of the E.CO.S.SE, $F(2,30) = 27.707$, $p < .001$. Two predictors (PEP-3-Expressive language and Vineland Adaptative Behaviour Scales, second edition – VABS-II –-Receptive language) accounted for 65% (simultaneous effect) of the variance in the scores of the E.CO.S.SE. Individually, the primary variable (PEP-3-Expressive language) explains 60%, and the secondary variable (VABS-II-Receptive language) explains 22% (simple effects).

Denomination-Phono 1 subtest: two-step regression model significantly predicted the scores of the Denomination-Phono 1 subtest, $F(2,26) = 21.476$, $p < .001$. Two predictors (PEP-3-Receptive language and VABS-II-Expressive language) accounted for 62% (simultaneous effect) of the variance in the scores of the Denomination-Phono 1 subtest. Individually, the primary variable (PEP-3-Receptive language) explains 55% and the secondary variable (VABS-II-Expressive language) explains 55% (simple effects).

Orofacial and lingual praxis subtest: one-step regression model significantly predicted the scores of the Orofacial and lingual praxis subtest, $F(1,27) = 12.165$, $p < .01$. One predictor (VABS-II-Receptive language) accounted for 31% of the variance in the scores of the Orofacial and lingual praxis subtest.

The analysis below addresses question (b): Do subdomains of hetero-assessments and developmental battery reflect the results of receptive and expressive standardized language tests? In this model, scores from the hetero-assessments and developmental batteries were entered as dependent variables, while Rasch scores from the standardized language tests served as predictor variables.

<u>Stepwise linear regression to explain receptive language scores from hetero-assessments and developmental batteries using Rasch scores from receptive language standardized tests</u>

Number of words understood in the French inventories of communicative development, or Inventaires Français du Développement Communicatif (IFDC)-12 months: no regression model predicts the number of words understood in the IFDC-12 months.

Number of words understood in the IFDC-18 months: no regression model predicts the number of words understood in the IFDC-18 months.

VABS-II-Receptive language: one-step regression model significantly predicted the scores of the Receptive language subdomain of the VABS-II, $F(1,37) = 8.924$, $p < .01$. One predictor (E.CO.S.SE) accounted for 19% of the variance in the scores of the Receptive language subdomain of the VABS-II.

PEP-3-Receptive language: one-step regression model significantly predicted the scores of the Receptive language subdomain of the PEP-3, $F(1,27) = 30.048$, $p < .001$. One predictor (E.CO.S.SE) accounted for 53% of the variance in the scores of the Receptive language subdomain of the PEP-3.

<u>Stepwise linear regression to explain expressive language scores from hetero-assessments and developmental battery using Rasch scores from expressive language standardized tests</u>

Number of words produced in the IFDC-12 months: no regression model predicts the number of words produced in the IFDC-12 months.

Number of words produced in the IFDC-18 months: no regression model predicts the number of words produced in the IFDC-18 months.

Number of words produced in the IFDC-24 months: no regression model predicts the number of words produced in the IFDC-24 months.

VABS-II-Expressive language: two-step regression model significantly predicted the scores of the Expressive language subdomain of the VABS-II, $F(2,36) = 34.623$, $p < .001$. Two predictors (Denomination-Lex 1 and Orofacial and lingual praxis subtests) accounted for 66% (simultaneous effect) of the variance in the scores of the Expressive language subdomain of the VABS-II. Individually, the primary variable (Denomination-Lex 1 subtest) explains 57%, and the secondary variable (Orofacial and lingual praxis subtest) explains 39% (simple effects).

PEP-3-Expressive language: two-step regression model significantly predicted the scores of the Expressive language subdomain of the PEP-3, $F(2,26) = 19.745$, $p < .001$. Two predictors (Denomination-Lex 1 and Orofacial and lingual praxis subtests) accounted for 60% (simultaneous effect) of the variance in the scores of the Expressive language subdomain of the PEP-3. Individually, the primary variable (Denomination-Lex 1 subtest) explains 52% and the secondary variable (Orofacial and lingual praxis subtest) explains 24% (simple effects).
